# Building a foundation for gene family analysis in Rosaceae genomes with a novel workflow: A case study in *Pyrus* architecture genes

**DOI:** 10.3389/fpls.2022.975942

**Published:** 2022-11-14

**Authors:** Huiting Zhang, Eric K. Wafula, Jon Eilers, Alex E. Harkess, Paula E. Ralph, Prakash Raj Timilsena, Claude W. dePamphilis, Jessica M. Waite, Loren A. Honaas

**Affiliations:** ^1^ Tree Fruit Research Laboratory, Agricultural Research Service (ARS), United States Department of Agriculture (USDA), Wenatchee, WA, United States; ^2^ Department of Horticulture, Washington State University, Pullman, WA, United States; ^3^ Department of Biology, The Pennsylvania State University, University Park, PA, United States; ^4^ College of Agriculture, Auburn University, Auburn, AL, United States; ^5^ HudsonAlpha Institute for Biotechnology, Huntsville, AL, United States

**Keywords:** tree architecture gene, gene family, comparative genomics, targeted genome re-annotation, European pear genome, Rosaceae, PlantTribes2

## Abstract

The rapid development of sequencing technologies has led to a deeper understanding of plant genomes. However, direct experimental evidence connecting genes to important agronomic traits is still lacking in most non-model plants. For instance, the genetic mechanisms underlying plant architecture are poorly understood in pome fruit trees, creating a major hurdle in developing new cultivars with desirable architecture, such as dwarfing rootstocks in European pear (*Pyrus communis*). An efficient way to identify genetic factors for important traits in non-model organisms can be to transfer knowledge across genomes. However, major obstacles exist, including complex evolutionary histories and variable quality and content of publicly available plant genomes. As researchers aim to link genes to traits of interest, these challenges can impede the transfer of experimental evidence across plant species, namely in the curation of high-quality, high-confidence gene models in an evolutionary context. Here we present a workflow using a collection of bioinformatic tools for the curation of deeply conserved gene families of interest across plant genomes. To study gene families involved in tree architecture in European pear and other rosaceous species, we used our workflow, plus a draft genome assembly and high-quality annotation of a second *P. communis* cultivar, ‘d’Anjou.’ Our comparative gene family approach revealed significant issues with the most recent ‘Bartlett’ genome - primarily thousands of missing genes due to methodological bias. After correcting assembly errors on a global scale in the ‘Bartlett’ genome, we used our workflow for targeted improvement of our genes of interest in both *P. communis* genomes, thus laying the groundwork for future functional studies in pear tree architecture. Further, our global gene family classification of 15 genomes across 6 genera provides a valuable and previously unavailable resource for the Rosaceae research community. With it, orthologs and other gene family members can be easily identified across any of the classified genomes. Importantly, our workflow can be easily adopted for any other plant genomes and gene families of interest.

## 1 Introduction

Advancements in plant genome sequencing and assembly have vigorously promoted research in non-model organisms. In horticultural species, new genome sequences are being released every month (Chen et al., 2021a; Chen et al., 2021b; Wang et al., 2021a, Wang et al., 2021b; Xu et al., 2021). These genomes have broadened our understanding of targeted cultivars and provided fundamental genomic resources for molecular breeding and more in-depth studies of economically important crop traits such as those involved in plant architecture. Although many gene families have been identified as important for architectural traits, such as dwarfing, weeping, and columnar growth (Hill and Hollender, 2019), the study of these genes and their functionality in new species is still hampered by inaccurate information about their gene models or domain structures, and the frequent lack of 1:1 orthology between related genes of different species. Sequencing and annotating a diversity of related genomes are crucial steps for obtaining this level of information.

Crops, most of which have gone through more than ten thousand years of domestication to meet human requirements, have a wide diversity in forms, sometimes even within the same species (Stansell and Björkman, 2020). One such example is in the *Brassica* species, where *B. rapa* encompasses morphologically diverse vegetables such as Chinese cabbage, turnips, and mizuna; and cabbage, stem kale, and Brussels sprouts are the same biological species, *B. oleracea*. Therefore, a single reference genome does not represent the complex genome landscape, or pan-genome, for a single crop species. To understand the genetic basis of the diverse *Brassica* morphotypes, many attempts have been made to explore the genomes of *Brassica* (Cheng et al., 2016a, Cheng et al., 2016b; Stansell et al., 2018; Stansell and Björkman, 2020; Mabry et al., 2021). In one of those attempts, genomes from 199 *B. rapa* and 119 *B. oleracea* accessions were sequenced and analyzed using a comparative genomic framework (Cheng et al., 2016a, Cheng et al., 2016b). Genomic selection signals and candidate genes were identified for traits associated with leaf-heading and tuber-forming morphotypes. Compared to *Brassica*, pome fruits may not appear to have as much diversity in their vegetative appearance, but they do have great diversity in terms of fruit quality, rootstock growth and performance, and post-harvest physiology. However, genome studies and pan-genome scale investigations in pome fruits are still in their infancy. In cultivated apple (*Malus domestica*), genomes of four different cultivars (Velasco et al., 2010; Daccord et al., 2017; Zhang et al., 2019; Sun et al., 2020b; Khan et al., 2022) have been published, providing resources to study: (1) small (SNPs and small InDels) and large scale (chromosome rearrangements) differences that can help explain cultivar diversity, and (2) gene content differences that may contribute to cultivar specific traits. However, genomic resources for European pear (*Pyrus communis*) cultivars are limited to just two published genomes (Chagné et al., 2014; Linsmith et al., 2019) from a single cultivar, ‘Bartlett’. More European pear genomes will afford new perspectives that help us understand shared and unique traits for important cultivars in *Pyrus*, as well as other Rosaceae.

Besides understanding large scale genomic characteristics, new genomes also provide rich resources for reverse genetic studies (Tollenaere et al., 2012; Wu et al., 2012). To obtain the actual sequence of a target gene, reverse genetic approaches in the pre-genome era relied on sequence and domain homology and technologies such as RACE PCR (Takos et al., 2006), which could be challenging and time consuming. Alternatively, in species with high-quality reference genomes, the annotation is generally considered to contain all the genes and target genes that could ideally be identified with a sequence similarity search (*i.e.*, BLAST). However, reports of annotation errors, such as imperfect gene models and missing functional genes are very common (Marx et al., 2016; Pertea et al., 2018; Pilkington et al., 2018). Another complicating factor is that duplication events (*i.e.*, whole genome duplication, regional tandem duplication) and polyploidy occur in the majority of flowering plants, including most crop species, posing substantial challenges to genome assembly and annotation (Kyriakidou et al., 2018). Moreover, instances of neofunctionalization and subfunctionalization occur frequently following duplication events (Hughes et al., 2014), which sometimes will result in large and complex gene families (Yang et al., 2015; Yoshida et al., 2019). Therefore, a one-to-one relationship between a gene in a model organism and its ortholog in other plant species, or even between closely related species and varieties, is rare (Xiao et al., 2013). Without understanding the orthology and paralogy between members of a given gene family, it is difficult to translate knowledge of a gene in a model organism to another species of interest.

In the present study, we assembled a draft genome for the European pear cultivar ‘d’Anjou’, improved the current ‘Bartlett’ assembly (*i.e.*, Bartlett.DH_V2), and developed a workflow that allows highly efficient target gene identification in any plant genome of interest. We used our workflow which iteratively curated and improved gene models for architecture-related genes from both the polished Bartlett.DH_v2 and the d’Anjou genomes. Importantly, we recovered many genes that were missing from gene families of interest (50 genes in the cultivar ‘Bartlett’) and corrected errors in others across the genus *Pyrus*. This work demonstrates that the integration of comparative genomics and phylogenomics can facilitate and enhance gene annotation, and thus gene discovery, in important plant reference genomes.

## 2 Materials and methods

### 2.1 Plant materials and sequencing

The ‘d’Anjou’ plants were purchased from Van Well’s nursery in East Wenatchee, WA, USA and grown in the USDA ARS greenhouse #6 at Wenatchee, WA, USA. Fresh leaves (~1.5g) from one ‘d’Anjou’ plant were flash frozen and used for DNA extraction. A CTAB isolation protocol (Michiels et al., 2003) was used to generate high-molecular-weight genomic DNA with the following modifications: the extraction was performed at large-scale with 100 ml of extraction buffer in a 250 ml Nalgene centrifuge bottle; the isopropanol precipitation was performed at room temperature (~ 5 minutes) followed immediately by centrifugation; after a 15-minute incubation in the first pellet wash solution, the pellet was transferred to a 50 ml centrifugation tube *via* sterile glass hook before performing the second pellet wash; following the second pellet wash, centrifugation, and air drying, the pellet was resuspended in 2 ml TE buffer (10 mM Tris, 1 mM EDTA, pH 8.0) and allowed to resuspend at 4°C overnight. The concentration of the DNA was measured by a Qubit 2.0 fluorometer (Invitrogen) and 50 ug DNA was digested with RNase A (Qiagen, final concentration 10 ug/ml, 37°C for 30 minutes) and then further cleaned up using the PacBio recommended, user-shared gDNA clean-up protocol (https://www.pacb.com/search/?q=user+shared+protocols) performed at large-scale with the DNA sample brought up to 2 ml with TE and all other volumes scaled up accordingly. The final pellet was resuspended in 100 ul TE. The final DNA concentration was measured by Qubit fluorometer, and 500 ng was loaded onto a PFG (Bio-Rad CHEF) to check the size range. The DNA ranged in size from 15 Kb to 100 Kb with a mean fragment size around 50 Kb. The purity of the DNA as measured by the NanoDrop spectrophotometer (ThermoFisher) was 260/280 nm: 1.91; 260/230 nm: 2.51. Cleaned-up gDNA was sent to the Penn State Genomics Core facility (University Park, PA, USA) for Pacbio and Illumina library construction and sequencing. A total of 10 ug gDNA was used to construct PacBio SMRTbell libraries and sequenced on a PacBio Sequel system. A small subset of the same gDNA was used to make Illumina TruSeq library and was sequenced on an Illumina HiSeq 2500 platform. In addition, 4 ug of the same gDNA was sent to the DNA technologies and Expression Analysis Core Laboratory at UC Davis (Davis, CA, USA) to construct an Illumina 10X Chromium library, which was sequenced on an Illumina NovaSeq 6000 sequencer.

### 2.2 Genome assembly and post-assembly processing

To create the initial backbone assembly of d’Anjou, Canu assembler v2.1.1 (Koren et al., 2017) was used to correct and trim PacBio continuous long reads (CLR) followed by a hybrid assembly of Illumina short reads and PacBio CLR with MaSuRCA assembler v3.3.2 (Zimin et al., 2013). Next, Supernova v2.1.1, the 10x Genomics *de novo* assembler (Weisenfeld et al., 2017), was used to assemble linked-reads at five different raw read coverage depths of approximately 50x, 59x, 67x, 78x, and 83x based on the kmer estimated genome size, and the resulting phased assembly graph was translated to produce two parallel pseudo-haplotype sequence representations of the genome. The Supernova assembler can only handle raw data between 30- to 85-fold coverage of the estimated genome size. Therefore, the muti-coverage assemblies provide an opportunity to capture most of the genome represented in the ~234-fold coverage sequenced 10x Chromium read data. One of the pseudo-haplotypes at each of the five coverages was used for subsequent meta-assembly construction to improve the backbone assembly, and the quality of which was assessed using a combination of assembly metrics, including (1) contig and scaffold contiguity (L50), (2) completeness of conserved land plants (embryophyta_odb10) benchmarking universal single-copy orthologs (BUSCO v5.2.2) (Manni et al., 2021), and (3) an assembly size closer to the expected d’Anjou haploid genome size. The backbone assembly was incrementally improved by bridging gaps and joining contigs with the Quickmerge program (Chakraborty et al., 2016) using contigs from the five primary Supernova assemblies in decreasing order of assembly quality. The resulting meta-assembly at each merging step was only retained if improvement in contiguity, completeness, and assembly size was observed.

Next, the long-distance information of DNA molecules provided in linked-reads was used to correct errors introduced in the meta-assembly during both the *de novo* and merging steps of the assembly process with Tigmint (Jackman et al., 2018) and ARCS (Yeo et al., 2017). Tigmint aligns linked-reads to an assembly to identify and correct potential errors and breaks. The improved assembly is then re-scaffolded into highly contiguous sequences with ARCS using the long-distance information contained in the linked-reads. To further improve the d’Anjou meta-assembly, trimmed paired-reads from both the short insert Illumina and 10x Chromium libraries were used to iteratively fill gaps between contigs using GapFiller v1.10 (Boetzer and Pirovano, 2012), and correct base errors and local misassemblies with Pilon v1.23 (Walker et al., 2014). The genome assembly process is illustrated in **Supplementary Figure 1**.

### 2.3 Pseudomolecule construction

Before constructing the chromosomal-scale pseudomolecules, extraneous DNA sequences present in meta-assembly were identified and excluded (**Supplementary Figure 1**). Megablast searches with e-value < 1e-10 was performed against the NCBI nucleotide collection database (nt), and then the best matching Megablast hits (max_target_seqs = 100) against the NCBI taxonomy database were queried to determine their taxonomic attributions. Assembly sequences with all their best-matching sequences not classified as embryophytes (land plants) were considered contaminants and discarded. A second iteration of Megablast searches of all the remaining sequences (embryophytes) was performed against the NCBI RefSeq plant organelles database to identify chloroplast and mitochondrion sequences. Assembly sequences with high similarity (> 80% identity; > 50% coverage) to plant organelle sequences were discarded (Yoshida et al., 2019; Hämälä et al., 2021). Finally, the remaining meta-assembly contigs and scaffolds were ordered and oriented into chromosomal-scale pseudomolecules with RaGOO (Alonge et al., 2019) using the *Pyrus communis* Bartlett.DH_v2 (Linsmith et al., 2019) reference chromosomes (**Supplementary Figure 1**).

### 2.4 Assembly validation

**Figure 1:**
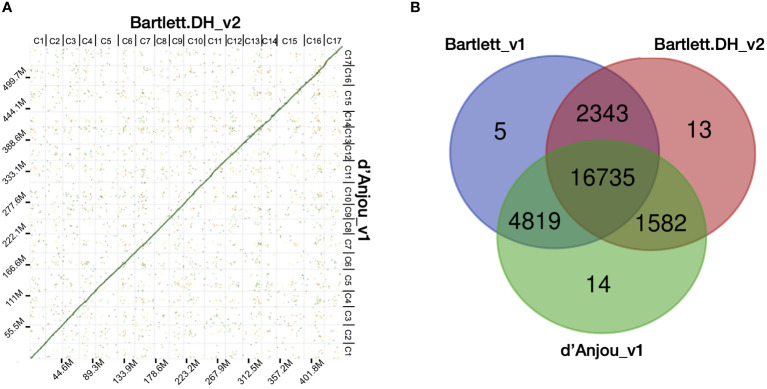
Characterization of the d’Anjou genome and protein orthology among European pears. **(A)** Dot plot of genome alignment of Bartlett.DH_v2 (x axis) and d’Anjou (y axis). **(B)** Overlap and distinctiveness of gene annotations among three *Pyrus communis* genotypes, Bartlett_v1, Bartlett.DH_v2, and d’Anjou.

The completeness of both the contig and scaffold assembly were evaluated by searching against the land plants (embryophyta_odb10) gene set with BUSCO v4 (Manni et al., 2021) (**Supplementary Table 1**). Synteny comparison between Bartlett.DH_v2 and d’Anjou meta-assembly were evaluated with D-GENIES (Cabanettes and Klopp, 2018) using repeat masked (http://www.repeatmasker.org) DNA alignments generated by minimap2 (Li et al., 2016b). Synteny results of the whole genome and each of the 17 *Pyrus communis* chromosomes are shown in **Figure 1** and **Supplementary Figure 2**, respectively.

### 2.5 Gene prediction

Prior to protein-coding gene annotation, we first estimated and masked the repetitive sequences in the d’Anjou meta-assembly following the protocol described by (Campbell et al., 2014). The meta-assembly was first searched using MITE-Hunter (Han and Wessler, 2010) and LTRharvest/LTRdigest (Ellinghaus et al., 2008; Steinbiss et al., 2009) to collect consensus miniature inverted-repeat transposable elements (MITEs) and long terminal repeat retrotransposons (LTRs), respectively. LTRs were filtered to remove false positives and elements with nested insertions. The cleaned LTRs were then used together with the MITEs to mask the genomes. The unmasked regions of the genomes were then annotated with RepeatModeler (http://www.repeatmasker.org/RepeatModeler) to predict additional *de novo* repetitive sequences. All collected repetitive sequences were compared to a BLAST database of plant proteins from SwissProt and RefSeq, and sequences with significant hits were excluded from the repeat masking library.

To supplement *ab initio* gene predictions, extensive extrinsic gene annotation homology evidence was collected, including (1) d’Anjou RNA-seq data from our previous study (Honaas et al., 2021); (2) homologous protein evidence of closely related species: *Malus domestica*, *Prunus persica*, *Pyrus betulifolia*, *Pyrus communis* ‘Bartlett’, *Pyrus* x *bretschneideri*, *Rosa chinensis*, and *Rubus occidentalis* retrieved from the Genome Database for Rosaceae (GDR) (Jung et al., 2018), and (3) protein sequences from the plant model species, *Arabidopsis thaliana* (Cheng et al., 2017).

Protein-coding gene annotations from the *Pyrus communis* reference genomes of Bartlett_v1 and Bartlett.DH_v2 were separately transferred (liftovers) to pseudomolecules of d’Anjou meta-assembly using the FLO (Pracana et al., 2017) (https://github.com/wurmlab/flo) pipeline based on the UCSC Genome Browser Kent-Toolkit (Kuhn et al., 2013). Next, repetitive and low complexity regions of the pseudomolecules were masked with RepeatMasker in the MAKER pipeline (release 3.01.02) (Cantarel et al., 2008) using the previously described d’Anjou-specific repeat library. Then, the MAKER pipeline updated the transferred annotations with gene annotation homology evidence (described above) and predicted additional protein coding genes with Augustus (Stanke et al., 2004; Hoff and Stanke, 2019) and SNAP (Korf, 2004). Only predicted gene models supported by annotation evidence, encode a Pfam domain, or both, were retained.

### 2.6 Computation of pear orthogroups

To compare the gene content of the three *Pyrus communis* genomes, Bartlett_v1, Bartlett.DH_v2, and d’Anjou, orthologous and paralogous protein clusters were estimated with OrthoFinder v1.1.8 (Emms and Kelly, 2015) from annotated proteins in all the genomes.

### 2.7 Bartlett.DH_v2 genome polishing

To improve the base quality of the publicly available pear reference genome, the *Pyrus communis* Bartlett.DH_v2 assembly was iteratively polished with two rounds of Pilon v1.24 (Walker et al., 2014) using the raw Illumina shotgun reads from the Bartlett.DH_v2 genome projects obtained from the NCBI Short Read Archive (SRA accessions: SRR10030340, SRR10030308), and completeness and accuracy assessed with the BUSCO v5.2.2 (Manni et al., 2021) embryophyta_odb10 database.

### 2.8 Gene family identification

Protein sequences of tree architecture candidate genes gleaned from published literature were sorted into pre-computed orthologous gene family clusters of 26 representative land-plant genomes (26Gv2.0) using the both BLASTp (Camacho et al., 2009) and HMMER hmmscan (Eddy, 2011) sequence search option of the *GeneFamilyClassifier* tool implemented in the PlantTribes 2 pipeline (https://github.com/dePamphilis/PlantTribes). Classification results of these architecture genes, including orthogroup taxa gene counts, corresponding superclusters (super orthogroups) at multiple clustering stringencies, and orthogroup-level annotations from multiple public biological functional databases are reported in **Supplementary Table 2**.

### 2.9 Gene family analysis

All the tools used in this process are modules from the command line version of PlantTribes 2 pipeline and are processed on SCINet (https://scinet.usda.gov/) with customized scripts **Supplementary File 8**. Protein coding genes from 14 Rosaceae genomes (*Fragaria vesca*, *Rosa chinensis*, *Rubus occidentalis*, *Prunus avium*, *Malus domestica* HFTH, *M. domestica* GDDH13, *M. domestica* Gala, *M. sieversii*, *M. sylvestris*, *Pyrus communis* Bartlett_v1, *Pyrus communis* Bartlett.DH, *Pyrus ussuriensis* x *communis*, *Pyrus bretschneideri*, *Pyrus communis* d’Anjou. Source of data and corresponding publications listed in **Supplementary Table 3**) were sorted into orthologous groups (26Gv2.0) with the *GeneFamilyClassifier* tool as previously described, after a quality control filtration using the *AssemblyPostProcessor* tool. A detailed summary of the Rosaceae gene family classification results are in **Supplementary Table 3**. Sequences classified into the orthogroups of interest (with candidate genes in this study) were integrated with scaffold backbone gene models using the *GeneFamilyIntegrator* tool. Gene names were modified as shown in **Supplementary Table 4** for easier recognition of the species and cultivar. Amino acid multiple sequence alignments and their corresponding DNA codon alignments were generated by the *GeneFamilyAligner* tool with the L-INS-i algorithm implemented in MAFFT (Katoh et al., 2002). Sites present in less than 10% of the aligned DNA sequences were removed with trimAL (Capella-Gutiérrez et al., 2009). Maximum likelihood (ML) phylogenetic trees were estimated from the trimmed DNA alignments using the RAxML algorithm (Stamatakis, 2014) option in the *GeneFamilyPhylogenyBuilder* tool. One hundred bootstrap replicates (unless otherwise indicated) were conducted for each tree to estimate the reliability of the branches. The multiple sequence alignments were visualized in the Geneious R9 software (Kearse et al., 2012) with Clustal color scheme. The phylogeny was colored with a custom script and visualized with Dendroscope version 3.7.5 (Huson and Scornavacca, 2012). Gene sequences, alignments, and phylogenies are available in **Supplementary Files 1–3**.

### 2.10 Domain prediction

To estimate domain structures of proteins in each orthogroup, the predicted amino acid sequences (either obtained from public databases or generated by the PlantTribes *AssemblyPostProcessor* tool) were submitted to interproscan v5.44-79.0 (Jones et al., 2014) on SCINet and searched against all the databases.

### 2.11 Targeted gene family annotation

The following approaches were used in parallel to annotate candidate genes from the original Bartlett.DH_v2, the polished Bartlett.DH_v2, and the d’Anjou genome assemblies:

#### 2.11.1 TGFam-finder

The ‘RESOURCE.config’ and ‘PROGRAM_PATH.config’ files were generated according to the author’s instruction. The three targeted genome assemblies mentioned above were used as the *target genomes*. Complete protein sequences from apples and pears in the same orthogroup were used as *protein for domain identification*. Complete protein sequences from other Rosaceae species and *Arabidopsis thaliana* in the same orthogroup were used as *resource proteins* for each annotation step. For each orthogroup, Pfam annotations from the InterProScan results were used as *TSV for domain identification*. For orthogroups without Pfam descriptions, MobiDBLite information was used as *TSV for domain identification* (Kim et al., 2020).

#### 2.11.2 Bitacora

Arabidopsis genes from targeted gene families (orthogroups of interest) were used to generate a multiple sequence alignment and HMM profile using MAFFT (Katoh et al., 2002) and hmmbuild (Eddy, 2011). The resulting files were then used as input for Bitacora v1.3, (Vizueta et al., 2020) running in both genome mode and full mode to identify genes of interest in the genome assemblies mentioned above.

### 2.12 Manual curation and gene model verification

In cases where both TGFam-Finder and Bitacora failed to predict a full-length gene, the gene model was curated manually.

#### 2.12.1 Curation with orthologous gene models

First, the genomic region containing the target sequence was determined either by the general feature format file (gff) or a BLASTn search using the coding sequence of the target gene or a closely related gene as a query. Next, a genomic fragment containing the target sequence and 3kb upstream and downstream of the targeted region was extracted. Then, the incomplete transcript(s), predicted exons, and complete gene models from a closely related species were mapped to the extracted genomic region using Geneious R9 (Kearse et al., 2012) with the *Map to Reference* function. The final gene model was determined by using the full-length coding sequence of a closely related gene as a reference.

#### 2.12.2 Curation with RNA-seq read mapping

The gff3 files obtained from Bitacora were loaded into an Apollo docker container v2.6.3 (Dunn et al., 2019) for verification of the predicted gene models using expression data. Publicly available RNA-seq data (Nham et al., 2015; Nham et al., 2017; Gabay et al., 2018; Zhang et al., 2018, Zhang et al., 2020; Hewitt et al., 2020) for *Pyrus* were used as inputs of an RNA-seq aligner, STAR v2.7.8a (Dobin et al., 2013), and alignments were performed with maximum intron size set to 5kb and default settings. Intron-exon structure was compared to the aligned expression data. If there was insufficient RNA-seq coverage from the targeted cultivar, data from other cultivars and *Pyrus* species were used as supporting evidence. Summaries of read mapping results are available in **Supplementary Files 4, 5**. Curated gene models from the original Bartlett.DH_v2 were transferred to the polished genome for validation.

Gene model cartoons were generated using the *visualize gene structure* function in TBtools v1.09854 (Chen et al., 2020). Final gene models and their corresponding chromosomal locations are available in **Supplementary Files 6, 7**.

## 3 Results

### 3.1 The draft d’Anjou genome

#### 3.1.1 Genome assembly

We generated approximately 134 million paired-end reads from Illumina HiSeq and a total of 1,054,992 PacBio continuous long reads (CLR) with a read length N50 of 20 Kb, providing an estimated 67-fold and 21-fold coverage respectively of the expected 600 Mb *Pyrus communis* genome (Chagné et al., 2014). Additionally, approximately 468 million 2 x 150 bp paired reads (~234-fold coverage) with an estimated mean molecule length (linked-reads) of 20 kb were generated using 10x Genomics Chromium Technology (**Supplementary Table 5**). The final meta-assembly, generated with a combination of the three datasets, contains 5,800 scaffolds with a N50 of 358 Kb (**Table 1**). The cleaned contigs and scaffolds were ordered and oriented into 17 pseudochromosomes guided by the reference genome, *Pyrus communis* ‘Bartlett.DH_v2’ (Linsmith et al., 2019).

**Table 1:** Comparison of genome assembly and annotation, and orthogroups among *Pyrus communis* genotypes.

Characteristics	Bartlett_v1	Bartlett.DH_v2	d’Anjou
Assembly			
Assembly size (Mb)	600	507.7	600
Number of scaffolds	142,083	592	5800
Scaffold N50	88 Kb	8.1 Mb	358.88 Kb
Pseudochromosomes	17	17	17
Complete BUSCOs	96.3%	98.3%	97.4%
Annotation			
Predicted gene number	43,419	37,445	45,981
Complete BUSCOs	93.1%	81.8%	92.9%
Mean CDS length	1209	1120	1343
Gene family classification			
Percentage of genes classified into pear orthogroups	76.2	76.2	80.4
Percentage of pear orthogroups containing genes	93.7	81	90.7
Number of 26Gv2 orthogroups containing genes	9878	9668	9837

Next, we compared the d’Anjou meta-assembly to two published reference assemblies of Bartlett (Chagné et al., 2014; Linsmith et al., 2019) to assess assembly contiguity, completeness, and structural accuracy. The Benchmarking Universal Single-Copy Ortholog (BUSCO) (Manni et al., 2021) analysis showed that the d’Anjou genome captured 97.4% complete genes in the embryophyta_odb10 gene sets, comparable to the reference genomes (**Table 1**; **Supplementary Table 6**). Furthermore, synteny comparisons between the draft d’Anjou genome and the reference Bartlett.DH_v2 genome showed high collinearities at both whole-genome and chromosomal levels (**Figure 1A**; **Supplementary Figure 2**).

#### 3.1.2 Annotation

Combining information such as *de novo* transcriptome assembly, homologous proteins of closely related species, and protein-coding gene annotations from the two ‘Bartlett’ genomes, we identified a total of 45,981 protein coding genes in d’Anjou (**Table 1**). Of those putative genes 76.63% were annotated with functional domains from Pfam (Mistry et al., 2020) and the remaining are supported by annotation evidence, primarily d’Anjou RNA-Seq reconstructed transcripts (Honaas et al., 2021). These results indicate that we captured a large majority of the gene space in the d’Anjou genome. This affords a range of analyses including gene and gene family characterization, plus global-scale comparisons with other Rosaceae species including the ‘Bartlett’ cultivar.

### 3.2 Comparison among three European pear genomes

To study the shared and genotype-specific genes among the three European pear genomes (Bartlett version1, Bartlett double haploid version 2, and d’Anjou version 1), we constructed 25,511 protein clusters (orthogroups), comprising 77.71% of all the genes. While numbers of predicted genes from the Bartlett_v1 and d’Anjou genomes may be overestimated due to the presence of alternative haplotype segments in the assembly caused by high heterozygosity (Linsmith et al., 2019), this should have very little effect on orthogroup circumscription. Further, the process of creating a double haploid reduces genome heterozygosity, but should retain estimates of orthogroup content. Hence, we formulated the following hypotheses: (1) a large majority of gene families are shared by all three genotypes; (2) few genotype-specific gene families are present in each genome; (3) the commercial ‘Bartlett’ genotype and the double haploid ‘Bartlett’ genotype (roughly version 1.0 and 2.0 of this genome, respectively) should have virtually identical gene family circumscriptions; and (4) we should detect very few gene families that are unique to either ‘Bartlett’ genome and shared with ‘d’Anjou’. The protein clustering analysis results (**Table 1**; **Figure 1B**) support our hypotheses 1 and 2: 65.60% of the orthogroups contain genes from all three genotypes and only 0.12% of the orthogroups are species-specific. However, among the 8,744 orthogroups containing genes from two genotypes, more than half (55.11%) are shared between d’Anjou and Bartlett_v1, 18.10% are shared by d’Anjou and Bartlett.DH_v2, and only 26.80% are shared between the two Bartlett genomes, which does not support hypotheses 3 and 4.

To better understand why these hypotheses lacked support, we took a broader look at gene family content by comparing a collection of Rosaceae genomes, including the pear genomes in question. We assigned all the predicted protein coding genes from 14 Rosaceae genomes of interest (Chagné et al., 2014; Daccord et al., 2017; Li et al., 2017; Shirasawa et al., 2017; Raymond et al., 2018; VanBuren et al., 2018; Xue et al., 2018; Linsmith et al., 2019; Ou et al., 2019; Zhang et al., 2019; Sun et al., 2020b) to orthogroups constructed with a 26-genome scaffold, covering most of the major lineages of land plants (**Supplementary Figure 3**). Out of the 18,110 orthogroups from this database, *Prunus persica*, a rosaceous species included in the genome scaffold, has representative genes in 10,290 orthogroups. Genes from most apple and pear genomes (Bartlett_v1, d’Anjou, *Malus domestica* HFTH_v1.0, *M. domestica* GDDH13_v1.1, *M. domestica* Gala_v1.0, *M. sieversii*_v1.0, *M. sylvestris*_v1.0) are present in more than 9,800 orthogroups, however, genes from Bartlett.DH_v2 were only found in 9,688 orthogroups (**Table 1**; **Supplementary Table 3**). These results suggest there are many genes not annotated in the Bartlett.DH_v2 genome.

### 3.3 Genome-wide identification of selected architecture genes

#### 3.3.1 A selection of architecture genes

With this new comparative genomic information, our next steps were two-fold: first, to leverage information from the three European pear genomes and other available Rosaceae genomes, to identify and improve a set of tree architecture-related gene models of interest, and second, to use these architecture gene families as a test case to investigate potential issues in the Bartlett.DH_v2 genome.

Many aspects of tree architecture are important for improving pear growth and maintenance, harvest, ripening, tree size and orchard modernization, disease resistance, and soil microbiome interaction. Traits of interest include dwarfing and dwarfism, root system architecture traits, and branching and branch growth. We selected key gene families known to be involved, particularly those that have been previously shown to influence architectural traits in fruit trees (**Supplementary Table 7**). The identification of genes within these families, as well as their genomic locations, correct gene models, and domain conservation, is an important early step in testing and understanding their relationships and functions.

#### 3.3.2 Overview of the gene dentification workflow

**Figure 2:**
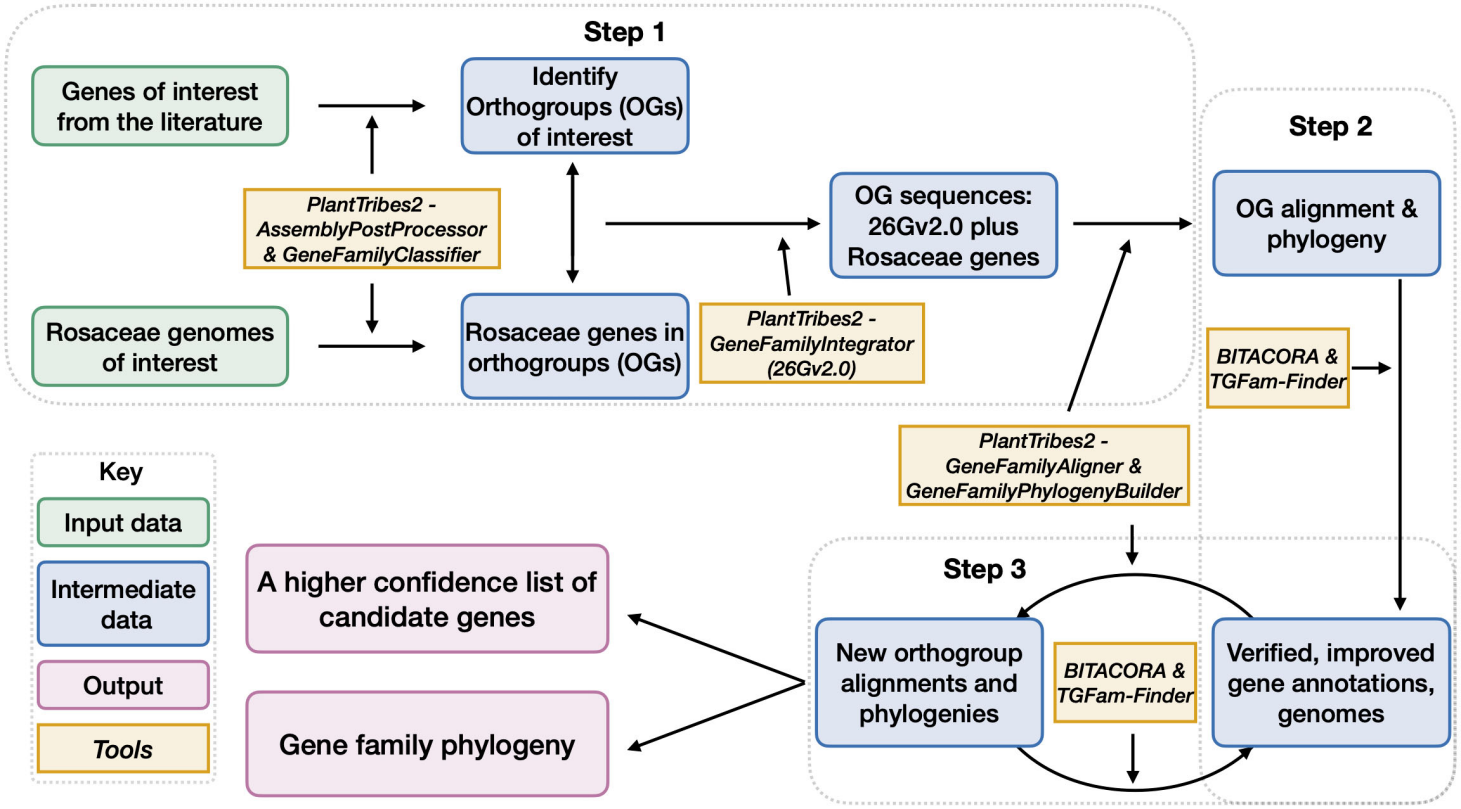
** **A workflow for candidate gene identification, curation, and gene family construction. Gray dotted boxes outlined the three steps of this workflow. Boxes with green outlines are input information. Boxes with blue outlines are intermediate outputs and boxes with purple outlines are final outputs. Contents in boxes with orange outlines are software used for generating the outputs.

Here, we developed a high throughput workflow (**Figure 2**), leveraging a subset of the best Rosaceae plant genomes and a phylogenomic perspective, to efficiently and accurately generate lists of genes in gene families of interest and phylogenetic relationships of genes from different plant lineages. Our workflow, consisting of three main steps, implemented various functions from PlantTribes2 (Wafula, 2019; https://github.com/dePamphilis/PlantTribes) and other software (Kim et al., 2020; Vizueta et al., 2020) for targeted gene annotation.

#### 3.3.3 Step 1 - An initial gene list and preliminary phylogenies

In Step 1, representative plant architecture genes obtained from the literature were assigned into orthogroups based on sequence similarity, giving us 22 orthogroups of interest (**Supplementary Tables 2**, **8**). Note that OG12636 is a monocot-specific orthogroup, thus not included in the downstream analysis of this section). We then leveraged the gene classification results of the aforementioned 14 Rosaceae genomes (**Supplementary Table 3**) and identified genes assigned to the 21 orthogroups of interest at a plant family level. Next, these Rosaceae genes were integrated with sequences from the 26 scaffolding species in the targeted 21 orthogroups for multiple sequence alignments, which were used to infer phylogeny. At the end of this step, we obtained our initial list of genes in each orthogroup and the phylogenetic relationship of genes in each gene family.

**Figure 3:**
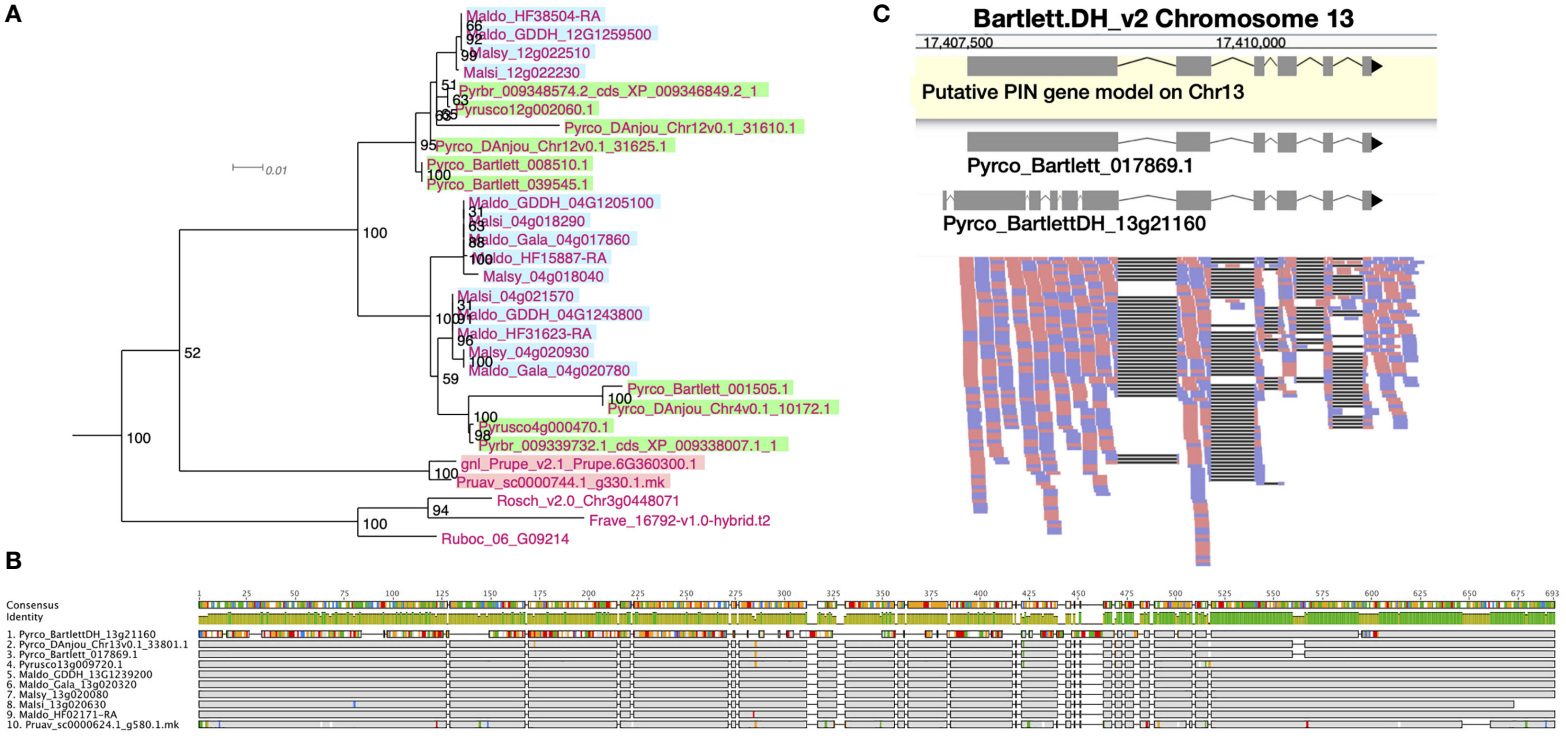
Phylogeny, amino acid sequence comparison, and RNAseq read mapping of *PIN* genes. **(A)** One clade of short *PINs* from OG1145 phylogeny. *Malus* genes are indicated with a blue background, *Pyrus* with a green background, and *Prunus* with a pink background. **(B)** Amino acid sequence alignment of orthologous genes from 10 Amygdaloideae species in the long *PIN* gene family (OG438). Sites identical to the consensus are shown in gray and sites different from the consensus are shown with a color following the Clustal color scheme in Geneious R9. Green color in the identity row indicates 100% identical across all sequences and greeny-brown color indicates identity from > 30% to < 100% identity. Gaps in the alignment are shown with a straight line. **(C)** RNAseq reads (forward: red; reverse: blue) mapped to a fragment of chromosome 13 in the Bartlett.DH_v2 genome, where a long *PIN* gene, *Pyrco_BartlettDH_13g21160*, was annotated. The gene model in the yellow box is a putative gene model predicted with RNAseq reads mapped to this region. The two gene models above the read mapping are retrieved from the original annotations of Bartlett_v1 (*Pyrco_Bartlett_017869.1*) and Bartlett.DH_v2 (*Pyrco_BartlettDH_13g21160*).

After examining the 21 orthogroups, we identified 64, 105, 94, and 53 genes from *Prunus persica*, Gala_v1, d’Anjou, and Bartlett.DH_v2, respectively (**Supplementary Table 9**). A whole genome duplication (WGD) event occurred in the common ancestor of *Malus* and *Pyrus* (Sun et al., 2020b), but was not shared with *Prunus.* Therefore, we expect to see an approximate 1:2 ratio in gene numbers in many cases, which explains fewer genes in *Prunus* compared to Gala_v1 and d’Anjou. However, the low gene count in Bartlett.DH_v2 was unexpected. For instance, we observed a clade within a PIN orthogroup (OG1145) comprised of short *PIN* genes (Křeček et al., 2009), which seemed to lack genes from the Bartlett.DH_v2 genome altogether (**Figure 3A**). One gene copy is found in *Prunus* and Rosoideae species, and two copies are found in most of the Maleae genomes, but none were identified in Bartlett.DH_v2. In addition, in the four genomes mentioned above, we found a number of problematic genes (**Supplementary Table 9**), for example genes that appeared shorter than all other orthologs or contained unexpected indels likely due to assembly or annotation errors.

#### 3.3.4 Step 2 and 3 - Iterative reannotation of problematic gene models

Inaccurate and missing gene models are common in any genome, especially in the early annotation versions (Marx et al., 2016; Pilkington et al., 2018). In model organisms, such as human, mouse (https://www.gencodegenes.org/), and *Arabidopsis* (https://www.arabidopsis.org/), gene annotations are continuously being improved using experimental evidence, improved data types (*e.g.* full-length RNA molecule sequencing), and both manual and computational curation. Building a better genome assembly is another way to detect additional genes. For instance, the BUSCO completeness score increased from 86.7% in the initial ‘Golden Delicious’ apple genome (Velasco et al., 2010) to 94.9% in the higher-quality GDDH13 genome (Daccord et al., 2017), indicating that the latter genome captured approximately 120 more conserved single-copy genes. Hence, we hypothesized that the potentially missing and problematic gene models we observed in the two European pears could be improved by: (1) using additional gene annotation approaches; and (2) searching against improved genome assemblies.

To test whether further gene annotation would improve problematic gene models, we moved forward to Step 2 of our workflow, using results from Step 1 as inputs. For each orthogroup containing problematic European pear genes (**Supplementary Table 9**), we used a subset of high-quality gene models from Rosids identified in Step 1 as inputs and re-annotated these gene families in the two pear genomes. After using a combination of annotation software and manual curation, we found a total of 98 genes from the d’Anjou genome, and reduced the number of problematic or incomplete genes from 34 to 3. In Bartlett.DH_v2, we identified 20 complete genes that were not annotated in the original genome and improved the sequences of 7 previously problematic genes. However, the total number of the selected architecture genes in Bartlett.DH_v2 (73 genes among which 15 were problematic or incomplete) was still notably lower than that of d’Anjou (98 with 3 incomplete genes) or Gala (105 with 15 being incomplete, see **Supplementary Table 9**). In Step 3, which involves iterative steps of phylogenetic analysis and targeted gene re-annotation, we added additional information such as the improved d’Anjou genes and RNA-seq datasets as new resources to annotate Bartlett.DH_v2 genes, but found no improvements in identifying unannotated genes or improving problematic models.

Results gathered after the first iteration of Step 3 supported our hypothesis that extra annotation steps could help improve imperfect gene models and identify missing genes in the two targeted European pear genomes. However, there were still about 30 genes potentially missing in Bartlett.DH_v2, which led us to test whether polishing the genome assembly would further improve problematic or missing gene models.

#### 3.3.5 Step 3 - adding Bartlett.DH_v2 genome polishing

The quality of genome assembly is affected by many factors, including sequencing depth, contig contiguity, and post-assembly polishing. Attempts to improve a presumably high-quality genome are time consuming, and may prove useless if the genome is already in good condition. To initially determine whether polishing the genome assembly would be useful, we first investigated the orthogroups with problematic Bartlett.DH_v2 genes to seek for evidence of assembly derived annotation issues. Indeed, in most cases where we failed to annotate a gene from presumably the correct genomic region, we observed unexpected indels while comparing the Bartlett.DH_v2 genome assembly to other pears (**Supplementary Figure 4**; **Supplementary Table 10**). Unexpected indels in the Bartlett.DH_v2 genome were associated with incorrect gene models as well. For example, **Figure 3B** shows a subset of amino acid sequence alignments for a specific member (*Pyrco_BartlettDH_13g21160*) of a PIN orthogroup (OG438) comprised of the long *PIN* genes (Křeček et al., 2009), in which the Bartlett.DH_v2 gene model shared low sequence identity with orthologs from other Maleae species and *Prunus*. To validate the identity of the problematic gene models, we leveraged RNAseq data from various resources (Nham et al., 2015; Nham et al., 2017; Gabay et al., 2018; Zhang et al., 2018; Hewitt et al., 2020; Zhang et al., 2020) and mapped them to the Bartlett.DH_v2 gene models. In most cases where a conflict was present between the pear consensus, for a given gene of interest, and the Bartlett.DH_v2 gene model, the reads supported the consensus (**Figure 3C**). The frequent occurrence of truncated and missing genes in the Bartlett.DH_v2 genome may be caused by assembly errors (*e.g.*, base call errors, adapter contamination) that create erroneous open reading frames. This observation provided us with the first piece of evidence that the differences in gene family content observed in the Bartlett.DH_v2 genome may not only be caused by misannotations, but also assembly issues.

To further test whether improvement to the genome assembly would allow us to capture the problematic and missing genes, we polished the Bartlett.DH_v2 genome with Illumina reads from the original publication (Linsmith et al., 2019). We identified 98.40% complete BUSCOs in the polished genome assembly, very similar to the original assembly (**Supplementary Table 6**), indicating that polishing did not remove BUSCO genes. Using the polished genome, we reiterated Step 3 of our workflow and annotated a total of 103 genes in our gene families of interest, with only two gene models being incomplete (**Supplementary Table 9**). This new result doubled the number of genes we identified from the original genome annotation and brought the expected gene number into parity with other pome fruit genomes. This supports our hypothesis that genes were missing due to methodological reasons, and in this case, due to assembly errors.

### 3.4 Curation of a challenging gene family: The IGT family

**Figure 4:**
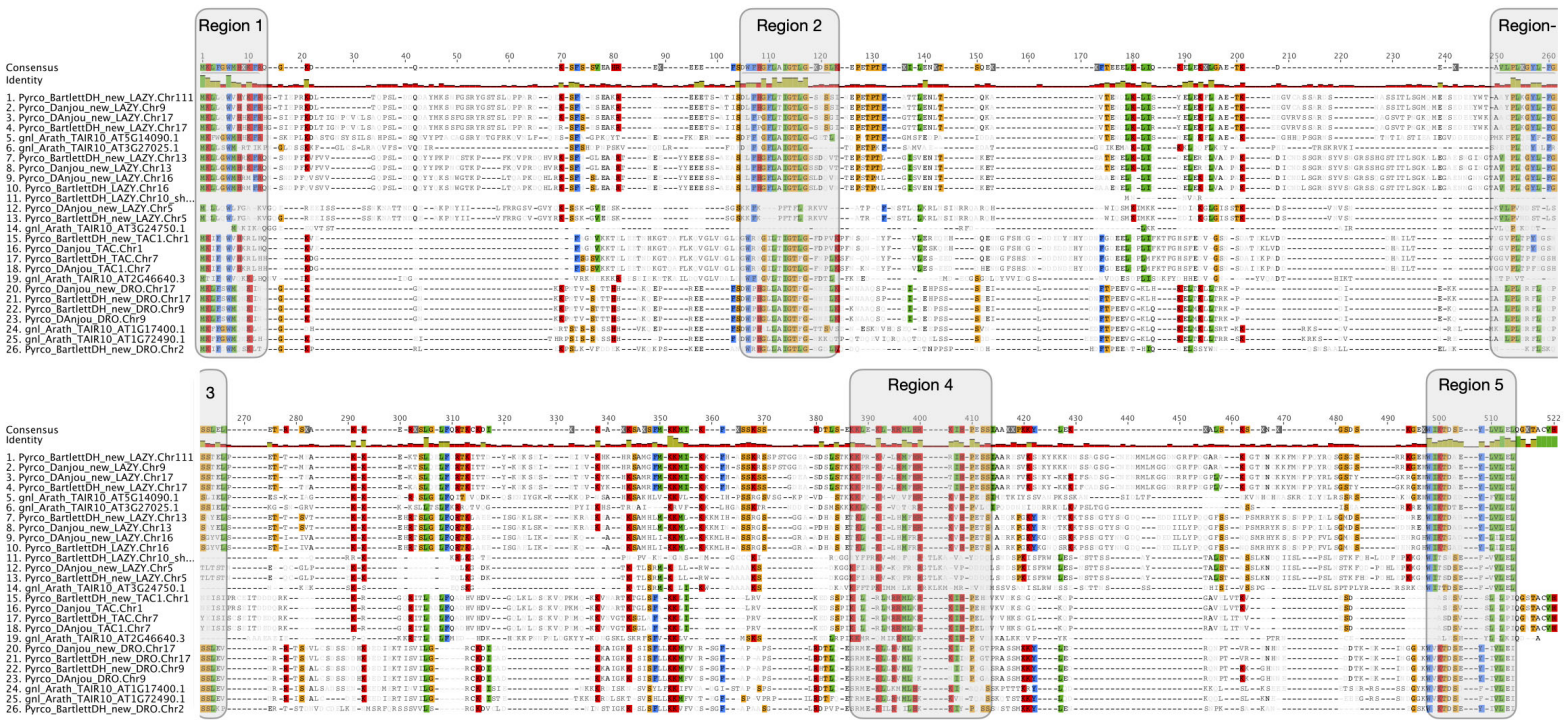
Amino acid comparison of *IGT* genes. Amino acid alignment of *IGT* genes from *Arabidopsis thaliana*, Bartlett.DH_v2, and d’Anjou. Sites consisting of a similar amino acid type as the consensus were highlighted with a background color following the Clustal color scheme in Geneious R9. Green color in the identity row indicates 100% identical across all sequences, greeny-brown color indicates identity from > 30% to < 100% identity, and red color indicates identity < 30%. Five conserved regions (domains) were highlighted with shaded boxes. Note: Region 3 is in both the upper and lower parts of the figure.

Some gene families are more complex than others. For example, it is more difficult to study the evolution of resistance (R) genes than most BUSCO genes because the former comprises fast-evolving multigene families while the latter are universally conserved single-copy gene families. Within the architecture gene families we studied, the IGT family is more challenging than many others because members of this family have relatively low levels of sequence conservation outside of a few conserved domains (Yoshihara et al., 2013). Previous reports identified four major clades (LAZY1-like, DRO1-like, TAC1-like, and LAZY5-like) in this gene family (Waite and Dardick, 2021). Study of LAZY1 in model species identified 5 conserved regions (Yoshihara et al., 2013) (**Figure 4**). The same domains are also present in other LAZY1-like and DRO1-like proteins and the first 4 domains are found in TAC1-like proteins across land plants (Yoshihara and Spalding, 2017). LAZY5-like, the function of which is largely unknown, has only domains I and V. Early research of the *TAC1-like* and *LAZY1-like IGT* genes identified these genes as grass-specific (Li et al., 2007; Yu et al., 2007), as BLAST searches failed to find homologs in other plant lineages.

**Figure 5:**
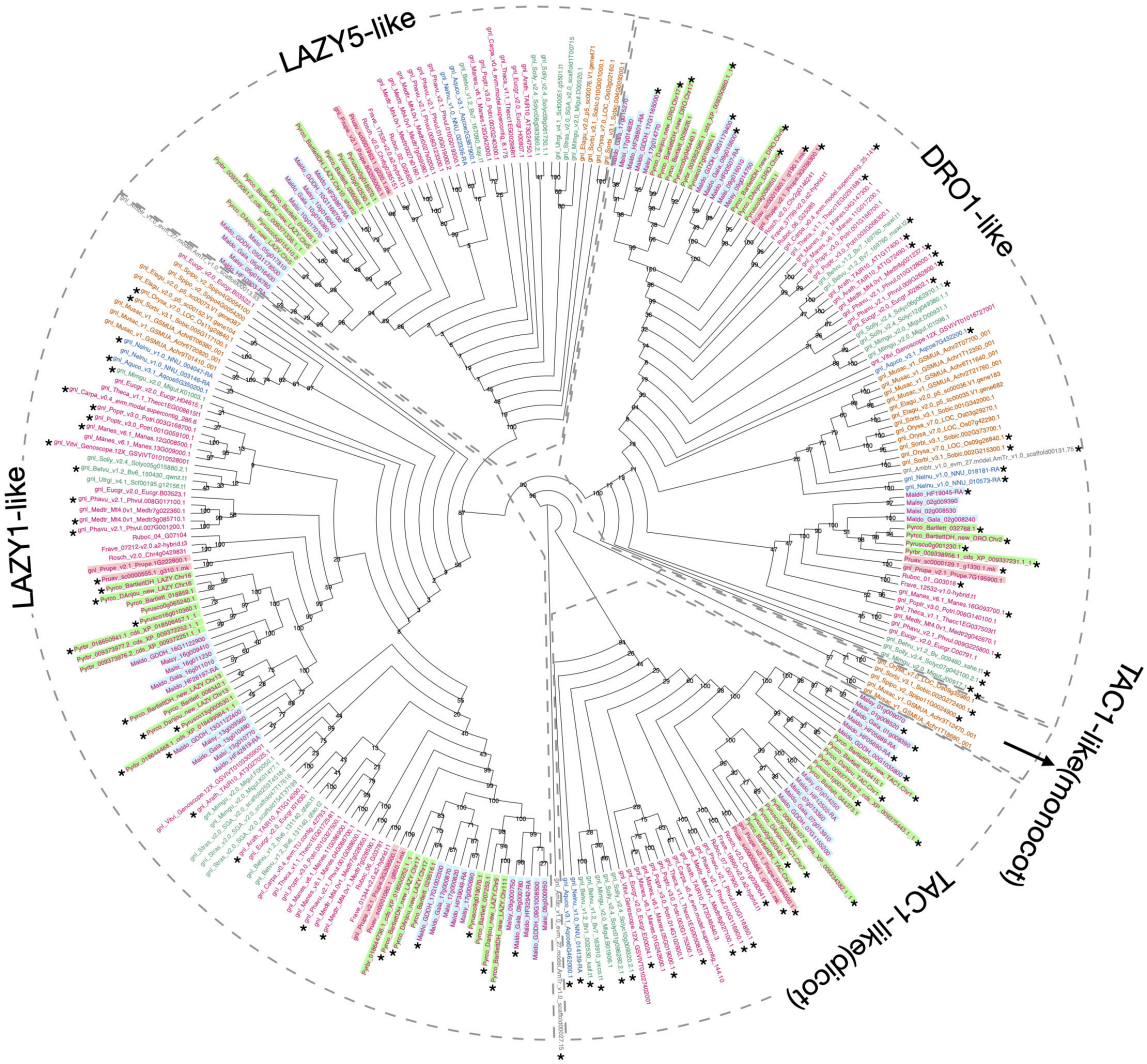
Phylogeny comparison of IGT genes. Cladogram of the IGT gene family (including LAZY1-like, LAZY5-like, TAC1-like, and DRO-like, separated by gray dotted boxes). The TAC1-like group was further divided into two monophyletic groups, with one containing only monocot genes and the other containing dicot genes. Genes are colored and highlighted as shown in **Supplementary Figure 3**. 1000 bootstrap replicates were conducted to estimate reliability and the numbers on the node indicate bootstrap support. Gene models with the expected domain structure and intron-exon structure were marked with *.

Using Arabidopsis and rice *IGT* genes as queries, our workflow identified five orthogroups (**Supplementary Table 2**), containing all the pre-characterized *IGT* genes in angiosperms. The phylogeny constructed with these five orthogroups largely supported previous classification of the four clades (Waite and Dardick, 2021), and provided more information regarding the evolutionary history of this gene family (**Figure 5**; **Supplementary Figure 5**). The TAC1-like clade, which is sister to the others, is divided into two monophyletic groups; one contains only monocots while the other has representatives from all the other angiosperm lineages. The LAZY1-like and LAZY5-like clades form one large monophyletic group, which is sister to the DRO1-like clade. Within Rosaceae, a near 1:2 ratio of gene number was expected between peach and pear due to the WGD in the common ancestor of the Maleae. Compared to the six known peach *IGT* genes (Waite and Dardick, 2021), we found 11 orthologs in Bartlett.DH_v2 (including 1 short gene, *Pycro_BartlettDH_LAZY.Chr10*, caused by an unexpected premature stop codon) and 9 in d’Anjou (*Pycro_Danjou_DRO.Chr2* and *Pycro_Danjou_LAZY.Chr10* failed to be annotated due to missing information in the genome). The resulting phylogeny (**Figure 5**) shows that we have now identified most of the expected *IGT* genes in European pears.

Besides low sequence similarity, *IGT* genes also have unique intron-exon arrangements, which are conserved across Arabidopsis and a few other plant species (Uga et al., 2013; Yoshihara et al., 2013; Waite and Dardick, 2021). These genes all contain 5 exons, but unlike most genes, the first exon only comprises six nucleotides and the last exon contains ~20 nucleotides. Annotation of short exons, especially when transcriptome evidence is limited, can be very challenging and skipping such exons could cause problems in gene discovery (Mount, 2000; Guo and Liu, 2015; Sharma et al., 2018). For instance, the annotation of *AtAPC11* (*At3g05870*) was inaccurate until Guo and Liu identified a single-nucleotide exon in this gene (Guo and Liu, 2015).

**Figure 6:**
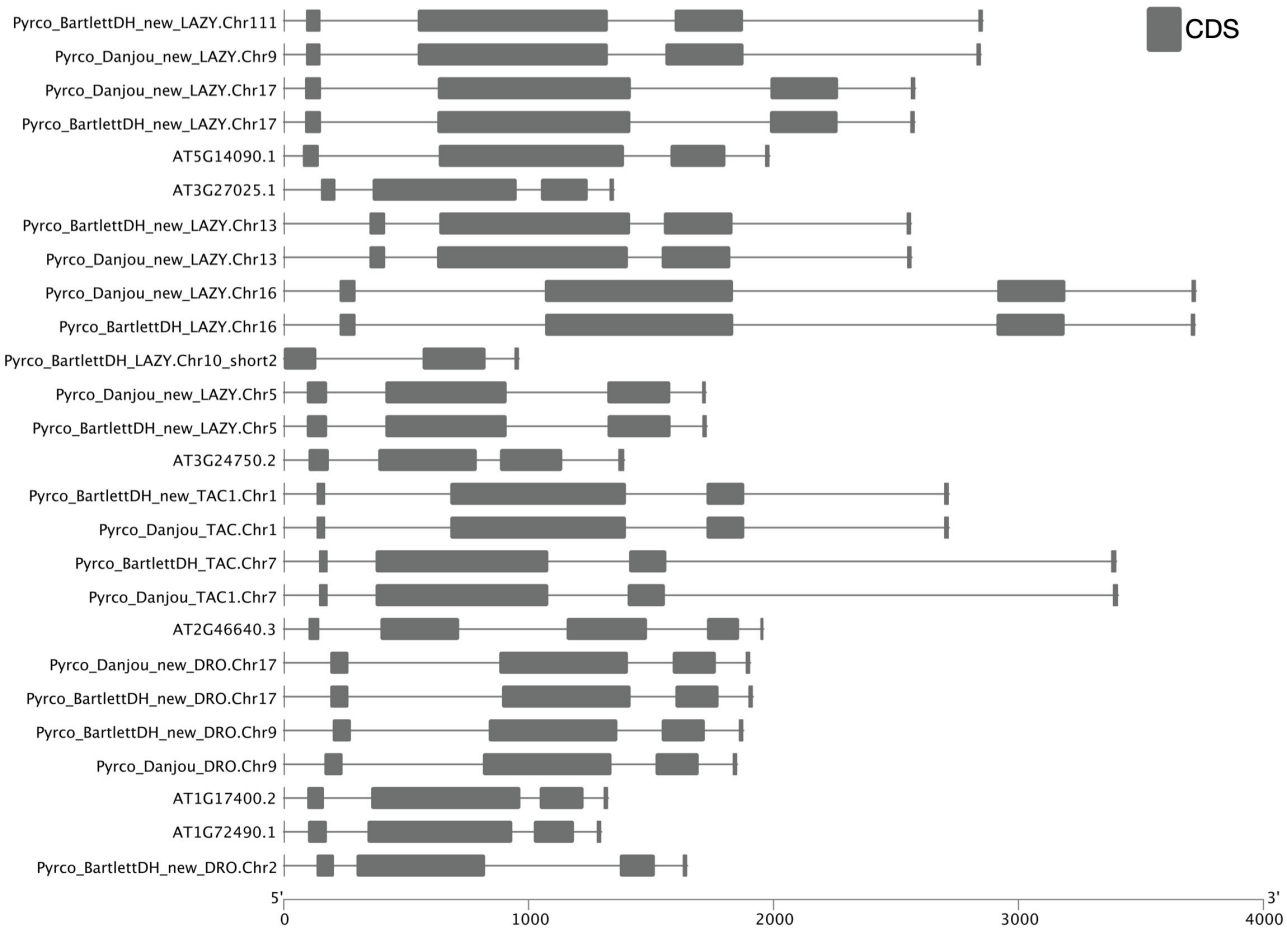
Intron-exon structure comparison of IGT genes. Cartoon illustrating intron-exon structures of IGT genes from *Arabidopsis thaliana* (Araport11), Bartlett.DH_v2, and d’Anjou. Boxes indicate exons, lines indicate introns. UTR regions are not shown in this figure.

To determine whether we captured the correct *IGT* gene models in the targeted genomes, we investigated the protein sequence alignments and gene features. In the original annotation, only three gene models (*Pyrco_BartlettDH_16g10510*, *Pyrco_BartlettDH_07g15250*, *Pyrco_DAnjou_Chr7v0.1_17442.1*) have the correct intron-exon combination and the expected domains. In the iterative re-annotation steps of our workflow, we identified 6 additional accurate gene models leveraging sequence orthology and transcriptome evidence. We further investigated all the sequences we identified as *IGT* genes, seeking the presence or absence of the expected domain features. However, even among gene models from the best annotated genomes used to construct the 26Gv2.0 database, only 45.16% (56/124) have the expected domain features (indicated with an * next to gene names in **Figure 5**. LAZY5-like was not taken into consideration due to its unique structure). In most cases, although the signature IGT domain (II) is correctly identified in the genes, domains I and V are usually missing or incorrect, likely due to misannotation of the first and last short exons. In Rosaceae, besides Bartlett.DH_v2 and d’Anjou, only 34.38% (33/96) had the expected domains (**Figure 5**). This finding motivated us to manually investigate the targeted genomes to annotate the IGT genes. Using the correct gene models as reference, plus a careful manual curation, we were able to annotate 19 complete gene models of the 20 expected IGT genes from the two targeted pear genomes (**Figures 4**, **6**).

## 4 Discussion

A second European pear cultivar genome from ‘d’Anjou’ provided additional insights into gene families across Rosaceae. By leveraging perspectives from comparative genomics and phylogenomics, we developed a high-throughput workflow using a collection of bioinformatic tools that takes a list of genes of interest from the literature and genomes of interest as input, and produces a curated list of the targeted genes in the query genomes.

In the case study presented here, candidate genes from 16 plant architecture-related gene families were identified from 15 Rosaceae genomes. The study of gene families consists primarily of two initial parts: first, identification of all the members in these families, and second, investigation of their phylogenetic relationships. Many attempts (Feng et al., 2019; Cancino-García et al., 2020; Zheng et al., 2020) to identify genes of interest from a genome have relied solely on a BLAST search querying a homolog from a model organism, which may be distantly related. However, such a method is insufficient in identifying all members of a large complex gene family or a fast-evolving and highly-divergent family, such as the *IGT* genes. They may also incorrectly include genes in a gene family based only on one or a few highly conserved regions that are insufficient for gene family membership. Compared to a BLAST-only approach, the gene classification process in our workflow used a combination of BLAST and HMMER search against an objectively pre-classified gene family scaffold, which provides a better result by taking into consideration both sensitivity and specificity (Wafula, 2019). This allowed us to efficiently identify even very challenging genes. Moreover, instead of selecting homologs based on simple statistics such as identity or bitscore, we took a phylogenetic approach and a sample dataset with references from a wide range of land plants to increase the accuracy of identifying orthologs and paralogs. Phylogenetic relationships revealed by a small number of taxa, for instance using only one species of interest and one model organism, can be inaccurate. For example, in our phylogenetic analysis with rich taxon sampling, *PIN5-1* and *PIN5-2* from *Pyrus bretschneideri* are sisters to all other *PINs* (**Supplementary Figure 6**), challenging the phylogenetic relationship inferred with *PINs* only from *P. bretschneideri* and *Arabidopsis thaliana* (Qi et al., 2020).

The iterative quality control steps in the workflow helped identify problems that existed in certain gene models and provided hints about where to make targeted improvements to important *Pyrus* genomic resources. The highly contiguous assembly of Bartlett.DH_v2 provided a valuable reference to anchor the shorter scaffolds from d’Anjou, which is essential for a good annotation. On the other hand, the perspective afforded by the d’Anjou genome led us to examine the Bartlett.DH_v2 genome assembly further. We developed and tested hypotheses regarding unexpected gene annotation patterns in the two targeted European pear genomes among various Maleae species and cultivars. This led to a polished assembly and improved annotations that allowed us to curate a high confidence list of candidate genes and gene models for downstream analyses. By adding targeted iterations of genome assembly and annotation, we now have a better starting point for reverse genetic analyses and understanding functionality of architecture-related genes in pears.

The challenges we encountered as we laid the groundwork for reverse genetics studies to understand pear architecture genes, and the approaches we took to evaluate and tackle these challenges, reinforce the idea that genome assembly and annotation are iterative processes. We found that relating gene accession IDs and inconsistent gene names back to gene sequences in various databases was often difficult and time consuming. Objective, global-scale gene classification, as we used here *via* PlantTribes2 (Wafula, 2019), can help researchers work across genomes and among various genome resources. Further, guidance from consortia such as AgBioData (Harper et al., 2018) is helping facilitate work such as we have described here that includes the acquisition and analysis of genome-scale data. Our starting point for understanding putative architecture genes in pear was with genes of interest from several plant species - an approach that many researchers will find familiar. With genes of interest in hand, our workflow provides a comparative genome approach to efficiently identify, investigate, and then improve and/or validate genes of interest across genomes and genome resources.

## Data availability statement

The datasets presented in this study can be found in online repositories. The names of the repository/repositories and accession number(s) can be found below: https://www.ncbi.nlm.nih.gov/, PRJNA762155.

## Author contributions

HZ, JW, LH conceived and designed the research. PR prepared gDNA for sequencing. HZ, EW, PT, JE, JW, CD, and AH performed the genome assembly and gene family analysis. All authors contributed to the article and approved the submitted version.

## Funding

This work was supported by the Washington Tree Fruit Research Commission project PR-17-104, the Agricultural Research Service in the US Department of Agriculture, and the Dottie and Lloyd Huck Endowment.

## Acknowledgments

The authors would like to acknowledge Heidi Hargarten for maintaining the d’Anjou plant and collecting leaf tissue for sequencing. They also thank Craig Praul at Penn State and Diana Burkart-Waco and Lutz Froenicke at UC Davis for sequencing.

## Conflict of interest

The authors declare that the research was conducted in the absence of any commercial or financial relationships that could be construed as a potential conflict of interest.

## Publisher’s note

All claims expressed in this article are solely those of the authors and do not necessarily represent those of their affiliated organizations, or those of the publisher, the editors and the reviewers. Any product that may be evaluated in this article, or claim that may be made by its manufacturer, is not guaranteed or endorsed by the publisher.
