# Supplementary material for: Building a foundation for gene family analysis in Rosaceae genomes with a novel workflow: A case study in Pyrus architecture genes
Source: Front Plant Sci. 2022 Nov 14;13:975942. doi: 10.3389/fpls.2022.975942 (PMC9702816; doi:10.3389/fpls.2022.975942)
Supplement: Supplementary file 2 [file DataSheet_2.pdf]

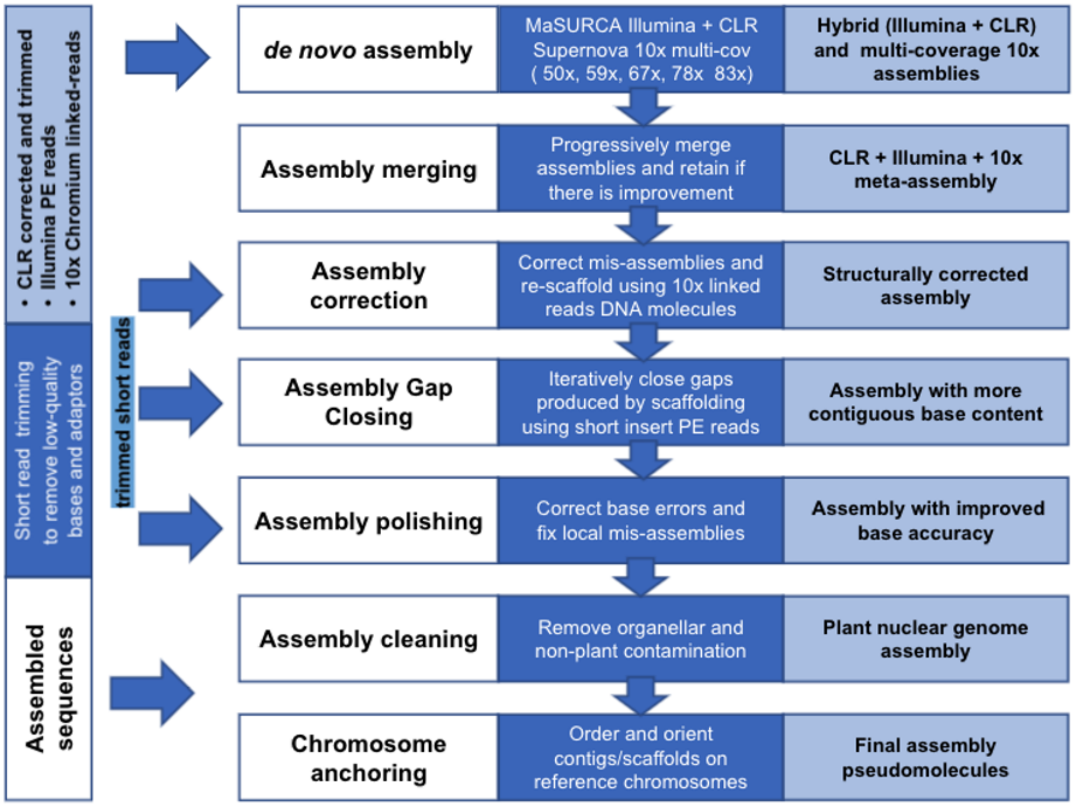

Supplementary Figure 1. A summary of the ‘d’Anjou’ genome assembly pipeline.

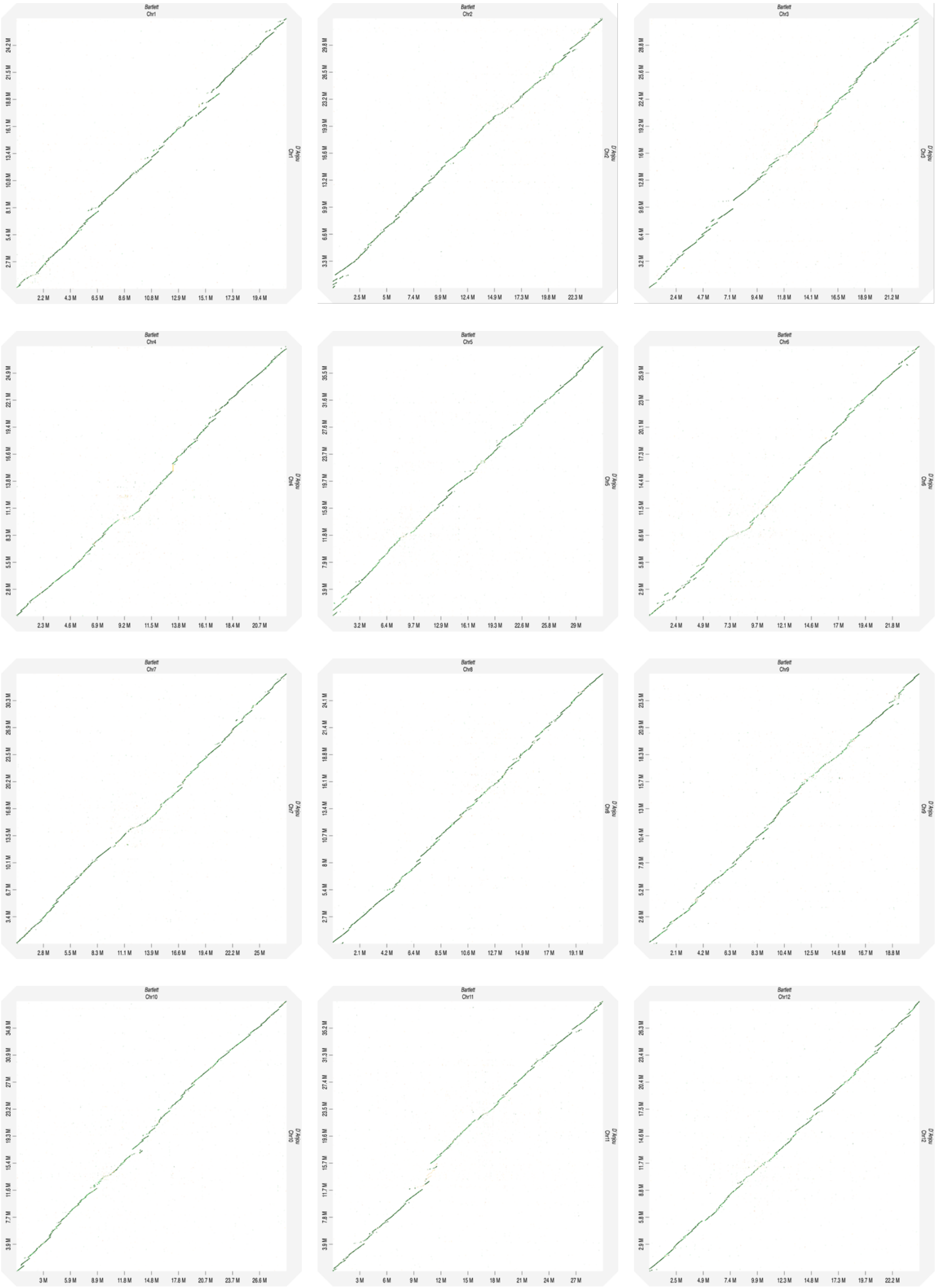

Bartlett
Chr1
D'Anjou
Chr1
2.7 M
5.4 M
8.1 M
10.8 M
13.4 M
16.1 M
18.8 M
21.5 M
24.2 M
2.2 M
4.3 M
6.5 M
8.6 M
10.8 M
12.9 M
15.1 M
17.3 M
19.4 M
Bartlett
Chr2
D'Anjou
Chr2
3.3 M
6.6 M
9.9 M
13.2 M
16.6 M
19.9 M
23.2 M
26.5 M
29.8 M
2.5 M
5 M
7.4 M
9.9 M
12.4 M
14.9 M
17.3 M
19.8 M
22.3 M
Bartlett
Chr3
D'Anjou
Chr3
3.2 M
6.4 M
9.6 M
12.8 M
16 M
19.2 M
22.4 M
25.6 M
28.8 M
2.4 M
4.7 M
7.1 M
9.4 M
11.8 M
14.1 M
16.5 M
18.9 M
21.2 M
Bartlett
Chr4
D'Anjou
Chr4
2.8 M
5.5 M
8.3 M
11.1 M
13.8 M
16.6 M
19.4 M
22.1 M
24.9 M
2.3 M
4.6 M
6.9 M
9.2 M
11.5 M
13.8 M
16.1 M
18.4 M
20.7 M
Bartlett
Chr5
D'Anjou
Chr5
3.9 M
7.9 M
11.8 M
15.8 M
19.7 M
23.7 M
27.6 M
31.6 M
35.5 M
3.2 M
6.4 M
9.7 M
12.9 M
16.1 M
19.3 M
22.6 M
25.8 M
29 M
Bartlett
Chr6
D'Anjou
Chr6
2.9 M
5.8 M
8.6 M
11.5 M
14.4 M
17.3 M
20.1 M
23 M
25.9 M
2.4 M
4.9 M
7.3 M
9.7 M
12.1 M
14.6 M
17 M
19.4 M
21.8 M
Bartlett
Chr7
D'Anjou
Chr7
3.4 M
6.7 M
10.1 M
13.5 M
16.8 M
20.2 M
23.5 M
26.9 M
30.3 M
2.8 M
5.5 M
8.3 M
11.1 M
13.9 M
16.6 M
19.4 M
22.2 M
25 M
Bartlett
Chr8
D'Anjou
Chr8
2.7 M
5.4 M
8 M
10.7 M
13.4 M
16.1 M
18.8 M
21.4 M
24.1 M
2.1 M
4.2 M
6.4 M
8.5 M
10.6 M
12.7 M
14.9 M
17 M
19.1 M
Bartlett
Chr9
D'Anjou
Chr9
2.6 M
5.2 M
7.8 M
10.4 M
13 M
15.7 M
18.3 M
20.9 M
23.5 M
2.1 M
4.2 M
6.3 M
8.3 M
10.4 M
12.5 M
14.6 M
16.7 M
18.8 M
Bartlett
Chr10
D'Anjou
Chr10
3.9 M
7.7 M
11.6 M
15.4 M
19.3 M
23.2 M
27 M
30.9 M
34.8 M
3 M
5.9 M
8.9 M
11.8 M
14.8 M
17.8 M
20.7 M
23.7 M
26.6 M
Bartlett
Chr11
D'Anjou
Chr11
3.9 M
7.8 M
11.7 M
15.7 M
19.6 M
23.5 M
27.4 M
31.3 M
35.2 M
3 M
6 M
9 M
12 M
15 M
18 M
21 M
24 M
27 M
Bartlett
Chr12
D'Anjou
Chr12
2.9 M
5.8 M
8.8 M
11.7 M
14.6 M
17.5 M
20.4 M
23.4 M
26.3 M
2.5 M
4.9 M
7.4 M
9.9 M
12.3 M
14.8 M
17.3 M
19.7 M
22.2 M

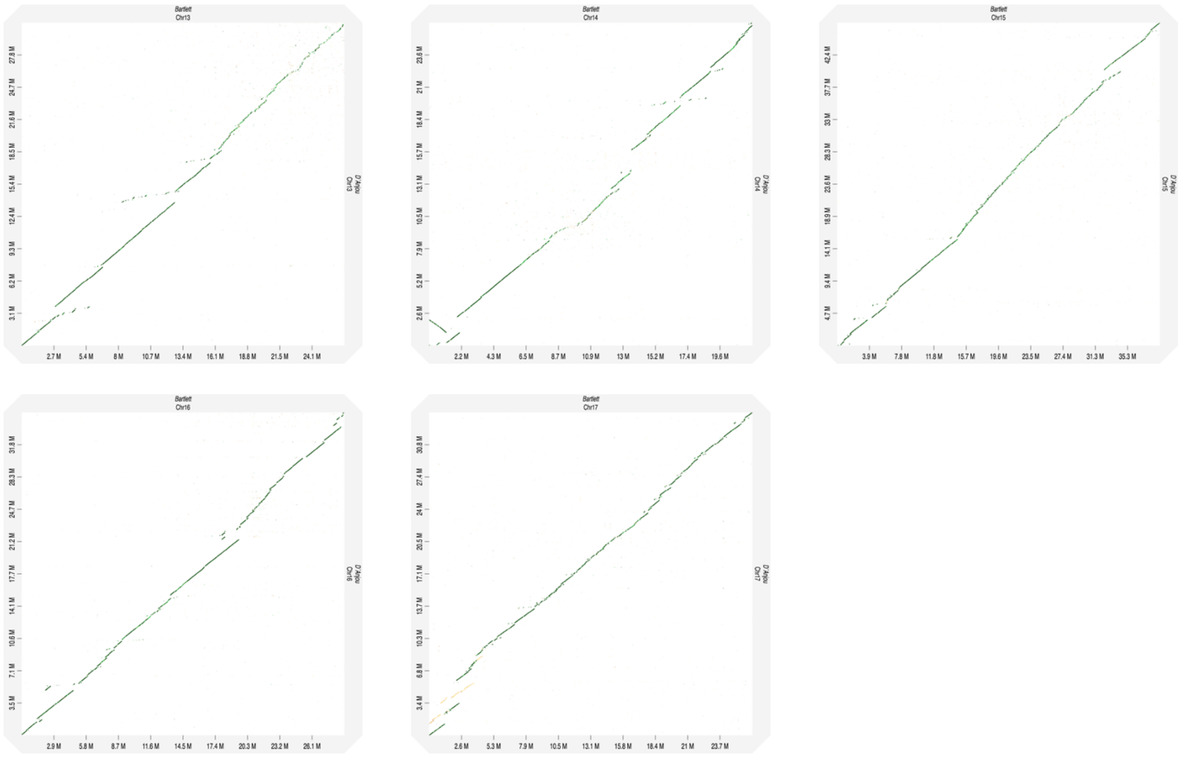

Supplementary Figure 2. Dot plot of chromosome alignments of Bartlett.DH_v2 (x axis) and d'Anjou_v1 (y axis).

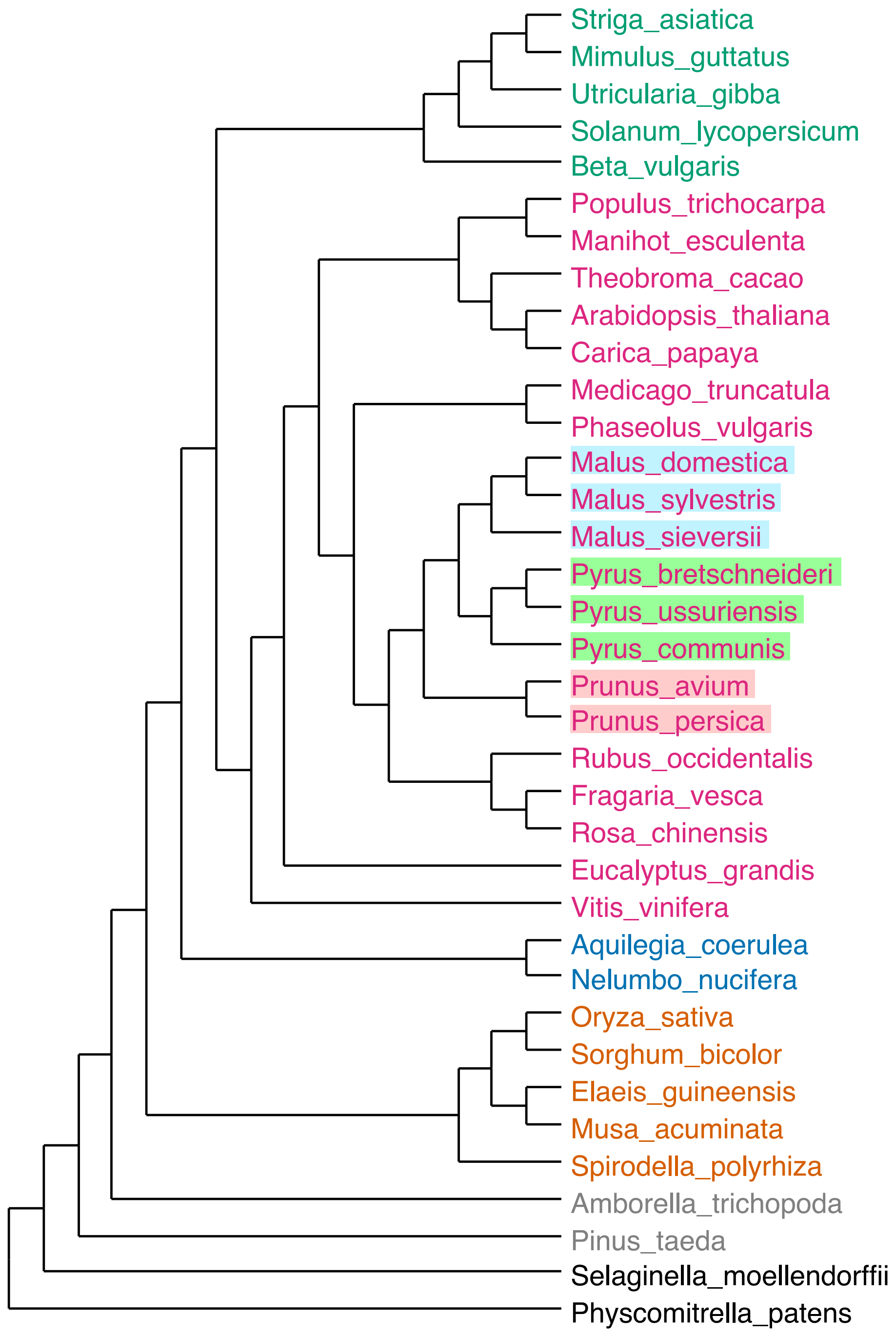

Supplementary Figure 3. A cladogram of the plant species used in this study. Rosaceae species except *Prunus persica* were classified into the 26 genome scaffold version 2. Genes from these species followed the same color scheme in the phylogenetic trees in this manuscript.

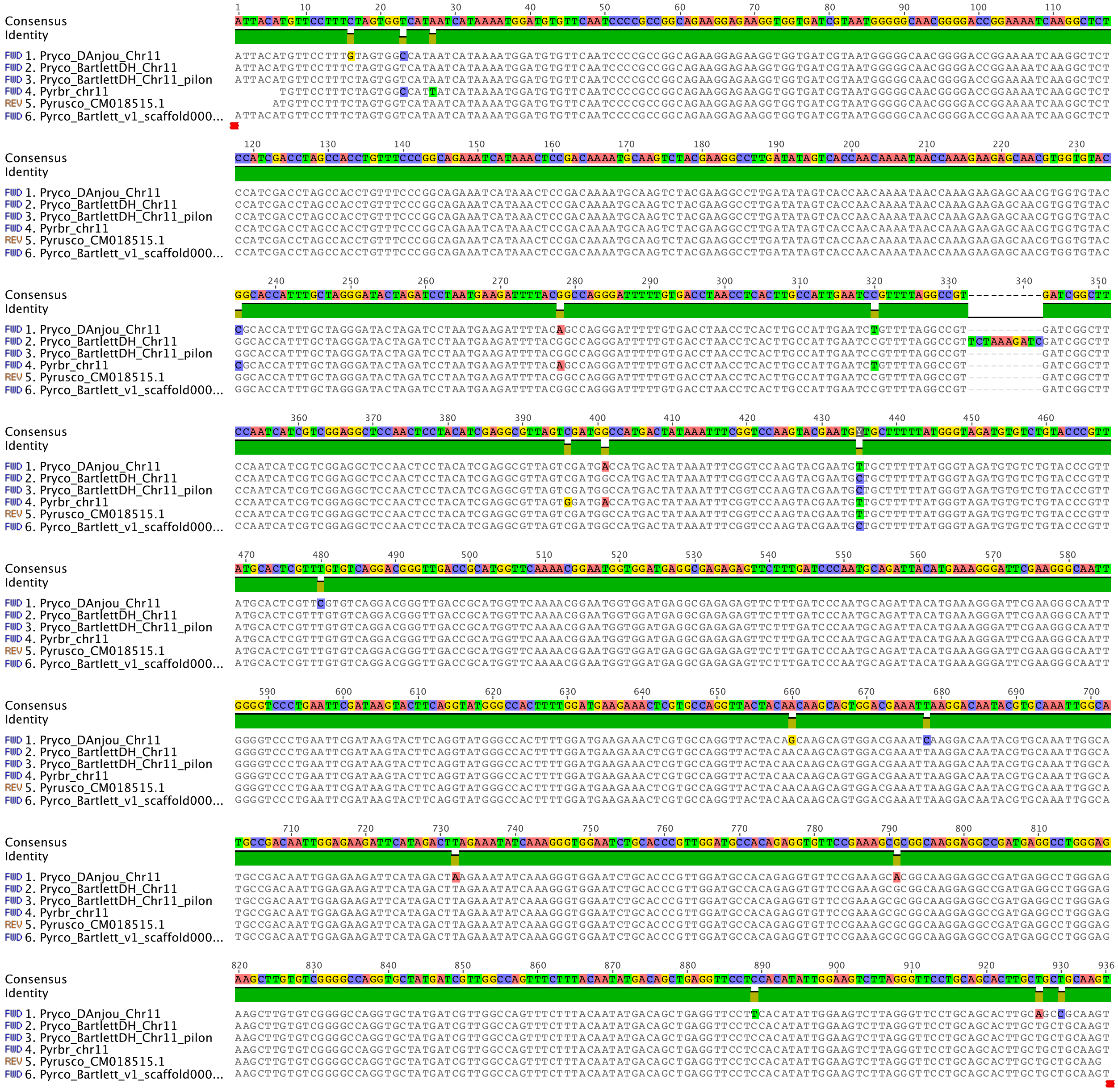

Supplementary Figure 4. Sequence comparison of genomic fragments where an *IPT* gene is expected to be located in various *Pyrus* genomes. Pyrco: *Pyrus communis*; Pyrbr: *P. bretschneideri*; Pyrusco: *P. ussuriensis x communis*. Sequence 2 is the original Bartlett.DH_v2 assembly and Sequence 3 is the polished assembly.

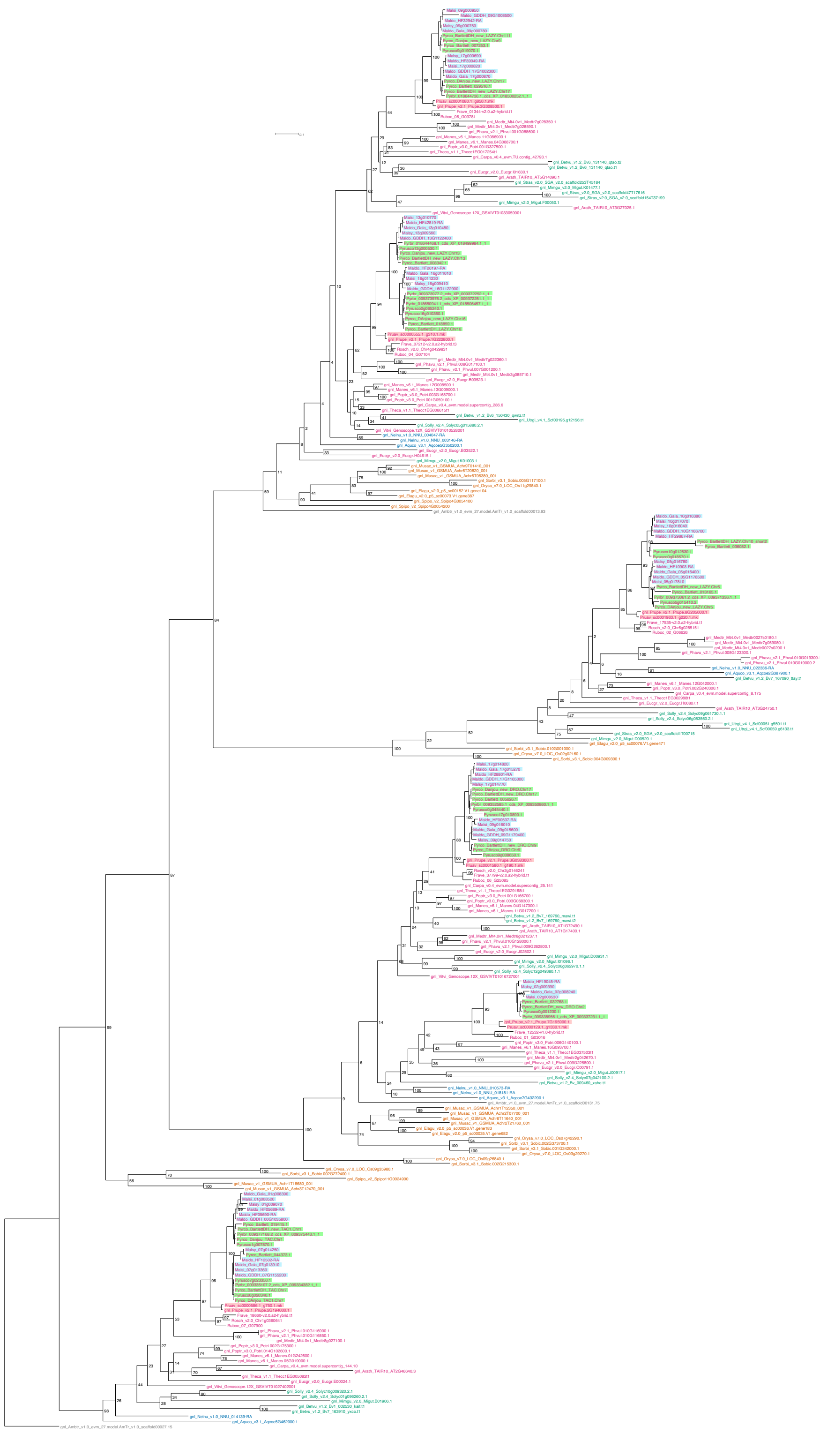

Supplementary Figure 5. Phylogram of the *IGT* gene family. Same phylogenetic tree as Figure 5.

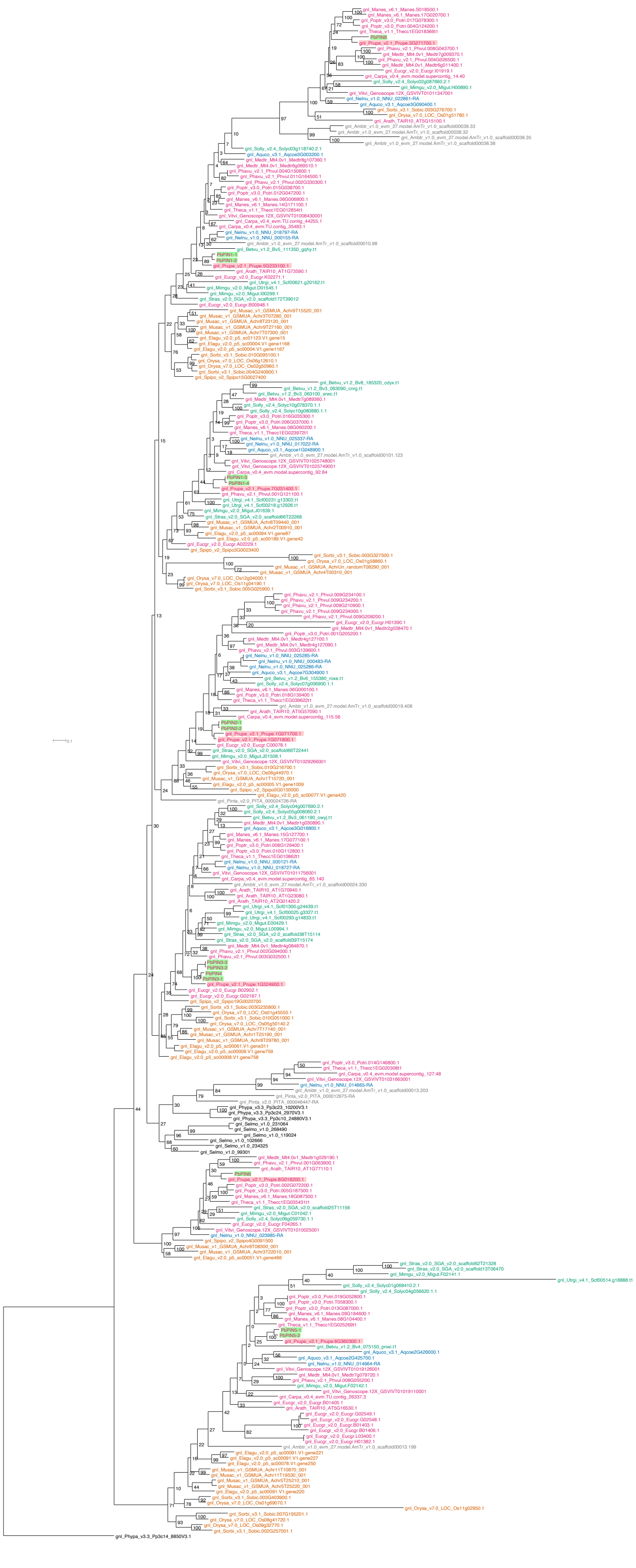

Supplementary Figure 6. Phylogeny of the *PIN* gene family inferred with the 26 genomes scaffold and *P. bretschneideri PIN* genes identified in reference Qi et al., 2020.
