# Supplementary material for: Building a foundation for gene family analysis in Rosaceae genomes with a novel workflow: A case study in Pyrus architecture genes
Source: Front Plant Sci. 2022 Nov 14;13:975942. doi: 10.3389/fpls.2022.975942 (PMC9702816; doi:10.3389/fpls.2022.975942)
Supplement: Supplementary file 4 [file Table_7.docx]

**Supplementary Table 7. Architecture gene family table**

| Family/  Gene | Species | Gene ID | Cultivar/  Ecotype | Method | Associated Architecture Phenotype | Refs |
| --- | --- | --- | --- | --- | --- | --- |
| PIN | |  |  |  |  |  |
| PIN1 | Apple (*Malus domestica*) | *MdPIN1*: *MDP0000138035* (*M. domestica* genome v1.0) | Royal Gala | Overexpression of *Malus PIN1* gene in Arabidopsis | Inhibition of primary root elongation, increased lateral root number, enhanced phototropic and geotropism responses. | An J. et al. (2017b) |
|  |  | *MdPIN1a*: *MF506847*  *MdPIN1b*: *MF506848*  (Genbank) | Rootstocks:  Baleng Crab, M9; Interstems:  M9;  Scion:  Red Fuji | Comparative gene expression between cultivars; subcellular localization; overexpression in tobacco; IAA quantification | Lower *MdPIN1b* expression in bark of M9 dwarfing rootstock and interstem; longer lateral roots, more adventitious roots, shorter and fewer root hairs in *MdPIN1b*-overexpressing tobacco lines. | Gan et al. (2018) |
|  | Pear (*Pyrus communis*, *P. bretschneideri*) | *PcPIN-L*: *PCP021016*  (*P. communis* genome v1.0) | Aihuali x Chili’ (*P. bretschneideri* Rehd.), Aihuali, Chili | Comparative gene expression between cultivars, across tissue types; subcellular localization; promoter activity; overexpression in tobacco, IAA quantification | Lower *PcPIN-L* expression in leaves, stems, roots, and seeds of pears exhibiting dwarfism; higher IAA content in shoot tips and lower IAA content in stems of pears exhibiting dwarfism; taller plants with longer cells in the stem, longer and more lateral roots in *PcPIN-L* overexpressing tobacco lines | Zheng, X. et al. (2018) |
| PIN1, PIN3 | Apple (*Malus robusta*, *M. spectabilis*) | *MrPIN1*: *MDP0000138035*  *MrPIN3*: *MDP0000497581* (*M. domestica* genome v1.0) | Rootstock:  *M. robusta*  Scion:  *M. specitabilis* Bly114 (Standard) and more-branching mutant (*MB*) | Gene expression (transcriptome and qPCR) and phenotypes of grafted plants, sugar and hormone quantification across tissues | The more-branching (*MB*) mutant repressed rootstock growth, and glycolysis and tricarboxylic acid activities. Rootstocks grafted with MB showed reduced *MrPIN1* expression, and increased *MrPIN3* expression. | Li, G. et al. (2016a) |
| PIN family | Pear (*Pyrus bretschneideri*, *P. betulifolia*, *P. communis*) | *PbPIN1-1*: *LOC103946937*  *PbPIN1-2*: *LOC103950573*  *PbPIN1-3*: *LOC103933990*  *PbPIN1-4*: *LOC103960490*  *PbPIN2-1*: *LOC103941631*  *PbPIN2-2*: *LOC103950477*  *PbPIN3-1*: *LOC103947028*  *PbPIN3-2*: *LOC103948593*  *PbPIN3-3*: *LOC103948670*  *PbPIN4*: *LOC103931858*  *PbPIN5-1*: *LOC103930394*  *PbPIN5-2*: *LOC103938552*  *PbPIN6*: *LOC103951142*  *PbPIN8*: *LOC103934837*  (Genbank) | Rootstock:  Douli  Interstems:  OHF51, QN101  Scion:  Xueqing | Comparative gene expression between cultivars | Compared *PbPIN* gene expression among different tissues of dwarfing OHF51 and vigorous QN101 rootstock cultivars, finding differential expression across tissues. Many *PbPINs* had higher shoot tip expression in the dwarfing rootstocks. | Qi et al. (2020) |
|  | Arabidopsis | *AtPIN1: At1g73590*  *AtPIN2: At5g57090*  *AtPIN3: At1g70940*  *AtPIN4: At2g01420*  *AtPIN5: At5g16530*  *AtPIN6: At2g77110*  *AtPIN7: At1g23080*  *AtPIN8: At5g15100*  (TAIR10) |  | Review of PIN protein evolution, protein structure, genomic structure, expression patterns, subcellular mechanisms, mutant phenotypes | Mutations in one or more *PIN* genes can lead to pin-like inflorescences, floral defects, gravitropism defects in the shoot or root, fused leaves or cotyledons, and loss of apical-basal patterning. | Křeček et al. (2009) |
|  | Across kingdom |  | All plants | Review of the role of auxin transport in branching forms across the plant kingdom | PIN proteins, via polar auxin transport, regulate branch initiation. branch outgrowth, and branch angle in flowering plants. | Harrison (2017) |
| AUX/LAX | |  |  |  |  |  |
| AUX1, LAX2 | Apple (*Malus domestica*) | *MdLAX2*: *MDP0000020317*, *MDP0000155074*  *MdAUX1*: *MDP0000155113*  (*M. domestica* genome v1.0) | Rootstock:  M9, M27, M793  Scion:  Royal Gala | Gene expression (transcriptome and qPCR) comparing dwarfing (M27, M9) and vigorous (M793) rootstocks, carbohydrate analysis | Dwarfing rootstocks exhibited a downregulation of *MdAUX1* and *MdLAX2* auxin transporters, among other differentially expressed genes. Further, starch synthesis was upregulated, and dwarfing rootstocks contained higher starch and lower fructose and glucose. | Foster et al. (2017) |
| AUX1 | Apple (*Malus domestica*) | *MdAUX1*: *MDP0000885425*  (*M. domestica* genome v1.0) | Hanfu (diploid and autotetraploid seedlings) | Phenotyping, Gene expression (Tag-seq and qPCR) comparing diploids and autotetraploids | Autotetraploid plants exhibited dwarfism. *AUX1* was found to be downregulated, in addition to changes in brassinosteroid gene expression (see below). | Ma et al. (2016) |
| AUX1/ LAX family | All plants (focus on models) | *AUX1/LAX* genes across species |  | Review | Auxin transport, via AUX1/LAX proteins, PINs, and PGP/ABCBs, plays a major role in many architecture-related developmental processes, such as root development (primary and lateral root, gravitropism, root hairs), phyllotactic patterning, leaf morphogenesis, and inflorescence architecture. | Swarup and Bhosale, 2019 |
| IGT | |  |  |  |  |  |
| TAC1 | *Prunus persica* | *PpeTAC1*: *Ppa010082*  (*P. persica* genome v1.0)  *AtTAC1*: *At2g46640*  (TAIR10) | Crimson Rocket | Pnome to map pillar mutation, gene expression, branch and flower bud angle phenotypes, *AtTAC1* overexpression in Arabidopsis | Pillar peach variety “Crimson Rocket” has insertion within *PpeTAC1* gene. Overexpression of *AtTAC1* in Arabidopsis leads to narrow branch angles. *PpeTAC1* and *AtTAC1* are expressed in branch tips. | Dardick et al. (2013) |
| TAC1, LAZY1, LAZY2 | Apple (*Malus domestica*) | *MdoTAC1a*: *MG837476*  *MdoTAC1b*: *MG837477*  MdoLAZY1: MG837478  *MdoLAZY2*: *MG837479*  (Genbank) | Rootstock:  *Malus robusta* Scion:  McIntosh, Wijcik, Granny Smith, Fukushima spur | Gene expression (qPCR) comparing cultivars representative of four architectural ideotypes. | Comparison of *IGT* gene expression between McIntosh (standard), Fukushima spur (spur), Granny Smith (tip-bearing), and Wijcik (columnar), showed decreasing levels of *TAC1* in shoot tips in that cultivar order, with the lowest expression of all identified *IGT* genes in the columnar cultivar in all tissues. | Wang, L. et al. (2018) |
| DRO1 | Apple (*Malus domestica*) | *MdDRO1*: *MDP0000142588*  *MdDRO2*: *MDP0000151294*  *MdDRO3*: *MDP0000723826* *MdPIN11*: *MDP0000125862*  (*M. domestica* genome v1.0) | Rootstock:  M9, Baleng Crab and interspecific hybrids from M9 x BC cross (rooted cuttings) | Root angle phenotyping, gene expression (qPCR in roots tissue), IAA quantification | Deep-rooted Baleng Crab had steeper root angles, and greater root length than M9. M9 showed lower IAA content, higher IAA oxidase activity, fewer amyloplasts, and lower expression of *MdDRO1* and *MdPIN11* architecture-associated genes. | An, H. et al. (2017a) |
|  | Plum (*Prunus domestica*),  Peach (*Prunus persica*),  Arabidopsis | *PpeDRO1*: *Ppa021925*  (*P. persica* genome v1.0)  *AtDRO1*: *At1g72490*  *AtDRO2*: *At1g19115*  *AtDRO3*: *At1g17400*  (TAIR10) | Plum seedlings:  Stanley  Arabidopsis ecotype:  Columbia-0 | Gene expression (qPCR, GUS), root angle phenotyping, root gravitropism, *DRO1* overexpression in plum and Arabidopsis | *DRO1* and *DRO2* are expressed in roots in Arabidopsis and peach. atdro1 mutants had wider lateral root angles, but no different in primary root gravitropism. *AtDRO1* OE Arabidopsis had narrower lateral root branch angles, distinct upward leaf curling, shorter siliques, narrower shoot branch angle. *PpeDRO1* OE plum roots were longer with greater root/shoot weight than controls. | Guseman et al., 2017 |
| IGT Family | Across kingdom | *IGT* gene across species |  | Review of *IGT* gene studies from the 1930’s to the present, phylogenetic analysis | *IGT* genes influence growth angle/gravitropic set point angle of both shoots and roots, which affects access to water and nutrients, density of plantings, structural integrity and soil anchorage, and overall crop productivity. | Waite and Dardick (2021) |
| GID1 | |  |  |  |  |  |
| GID1c | Peach (*Prunus persica*) | *PpeGID1c*: *Ppa018174*  (*P. persica* genome v1.0) | Brachytic dwarf (BD), Standard, and a mapping population from a BD x Std cross | Pnome to map *dw* mutation, phenotyping and GA application response, RNAi silencing of *GID1c* in plum, gene expression (qPCR) | BD peaches exhibit extreme dwarfism, primarily attributed to reduced internode length. The *dw* mutation in BD peaches was mapped to *PpeGID1c*. BD peaches showed insensitivity to GA treatment. Silencing *GID1c* in plums led to a BD-like dwarfed phenotype. | Hollender et al. (2016) |
|  |  |  | FenHuaShouXingTao (FHSXT), QiuMiHong (QMH) | Phenotyping of dwarf (FHSXT) and Standard (QMH) cultivars, GA response and quantification, gene expression (qPCR), yeast-2-hybrid, protein quantification (western) | FHSXT exhibited extreme dwarfism, short internodes, shorter cell length, fewer branches, and longer leaves. FHSXT had high levels of GA and were GA insensitive. *GID1c* was upregulated in FHSXT, as were multiple GA biosynthesis genes (see below). The mutation in FHSXT *GID1c* abolished interaction with DELLA1 in a yeast-2-hybrid assay. | Cheng et al. (2019) |
| GID1 | Arabidopsis | *AtGID1a*: *At3g05120*,  *AtGID1b*: *At3g63010*,  *AtGID1c*: *At5g27320*  (TAIR10) | Columbia-0 | Phenotyping mutants, gene expression (qPCR), GA treatment and quantification | Double mutants *atgid1a* *atgid1c* and *atgid1a* *atgid1b* showed reduced stem height phenotypes, while the triple mutant exhibited severe dwarfing and GA insensitivity. The triple mutant phenotype was partially rescued by loss of function of the DELLA protein AtRGA. | Griffiths et al. (2006) |
| GA2ox, GA3ox, GA20ox | | |  |  |  |  |
| GA2ox | Peach (*Prunus persica*) | *PpeGA2ox*-*1*: *Prupe.1G111900*  *PpeGA2ox-2*: *Prupe.1G344000*  *PpeGA2ox-3*: *Prupe.3G006700*  *PpeGA2ox-4*: *Prupe.4G026300*  *PpeGA2ox-5*: *Prupe.4G080700*  *PpeGA2ox-6*: *Prupe.4G150200*  *PpeGA2ox-7*: *Prupe.4G204600*  (*P. persica* genome v2.0) | QiuMiHong (QMH) | Gene expression (qPCR), PpeGA2ox gene overexpression in tobacco, GA treatment and quantification | Seven *GA2ox* genes were identified in peach and classified into three subgroups, and tissue-specific expression was determined in a standard peach cultivar. Overexpression of *PpeGA2ox1*, *PpeGA2ox5*, and *PpeGA2ox2* resulted in dwarf phenotypes in tobacco. GA treatment at shoot tips induced expressions of all *PpeGA2ox* genes, but at different rates. | Cheng et al. (2021) |
|  | *Arabidopsis thaliana* | *AtGA2ox1*: *At1g78440*  *AtGA2ox2*: *At1g30040*  *AtGA2ox3*: *At2g34555*  *AtGA2ox4*: *At1g47990*  *AtGA2ox6*: *At1g02400*  *AtGA2ox7*: *At1g50960*  *AtGA2ox8*: *At4g21200*  (TAIR10) | Columbia-0 and *hy5* mutant | Gene expression (GUS reporters, qPCR), *GA2ox* overexpression | Overexpression of *GA2ox* genes inhibited elongation of the hypocotyl, rescued the long-hypocotyl phenotype of hy5 mutants. Overexpression of *GA2ox7* and 8 led to extra shortening of the hypocotyl in *hy5* mutants. | Li et al. (2019) |
| GA20ox | Apple (*Malus domestica*) | *MpGA20ox1A*  *MpGA20ox1B*  (Noted from *M. pumila*, a previous name for *M. domestica*. Gene IDs unclear) | Rootstock:  M25, MM106  Scion:  Greensleeves | Gene expression (qPCR), gene silencing, phenotyping, grafting onto invigorating rootstocks, GA quantification | Silencing of *MpGA20ox1A* and *B* led to reduced height, and reduced internode length and number. Application of exogenous GA rescued the dwarfed phenotype. Transgenic dwarfed scions remained dwarfed after grafting onto invigorating rootstocks. | Bulley et al. (2005) |
| GA2ox, GA3ox, GA20ox | Peach (*Prunus persica*) |  | FenHuaShouXingTao (FHSXT), QiuMiHong (QMH) | See above (*GID1*) | Expression of several *GA2ox*, *GA3ox*, and *GA20ox* GA biosynthesis genes were upregulated in a dwarfed variety (FHSXT), as was *GID1c* (see above). | Cheng et al. (2019) |
| WRKY9 | |  |  |  |  |  |
| WRKY9 | Apple (*Malus domestica*) | *MdWRKY9*: *MDP0000272940*  (*M. domestica* genome v1.0) | Dwarfing rootstock:  M.9 Pajam 2, M26, GM256, B9 and M.9 T337  Non-dwarfing rootstock:  *M. baccata*, *M. robusta*, *M. sieversii*, *M. prunifolia*, *M. zumi* | Gene expression (qPCR), *MdWRKY9* overexpression in apple, subcellular localization, transcriptional inhibition assays, hormone quantification, CHIP-qPCR | *MdWRKY9* was more highly expressed in dwarfing rootstock. Overexpression of *MdWRKY9* in M26 semi-dwarfing rootstock resulted in dwarfed characteristics, and fewer, but longer roots. *MdWRKY9* binds and inhibits expression of the brassinosteroid biosynthetic gene *MdDWF4* (see below) | Zheng, X. et al. (2018) |
| EIN2 | |  |  |  |  |  |
| EIN2 | Rice (*Oryza sativa*) | *OsEIN2*: *LOC_Os07g06130*  *OsEIL1*: *LOC_Os03g20790*  (MSU Rice Gene Models) | Nipponbare | Computed tomography (CT), mutant analysis, confocal microscopy, ethylene treatment and quantification, gas barrier treatment, GUS assay, ethylene biosensor | Ethylene-insensitive signaling mutants (*osein2* and *oseil1*) were unaffected by and grew deeper into compacted soil. Shoot and root biomass was increased in ethylene-insensitive mutants grown in compacted soil. Ethylene was slow to diffuse through compacted soils and ethylene detection in the root was shown to increase in compacted soil. | Pandey et al. (2021) |
|  | *Arabidopsis thaliana* | *AtEIN2*: *At5g03280*  (TAIR10) | Columbia-0 | Gene expression (GUS), root architecture phenotyping on differing Boron concentrations, mutant analysis | Ethylene-insensitive mutant *atein2-1* inhibited increased root hair formation and elongation under low-Boron conditions. | Martín‐Rejano et al. (2011) |
|  | All species | Ethylene signaling and biosynthesis genes |  | Review of ethylene effects on root and hypocotyl elongation, root hair formation, apical hook formation, stem growth, biosynthesis pathway, and signaling pathway | Inhibitory effects of ethylene on root growth observed as early as 1901. Plant grown on aerated soils have high ethylene and strong root growth inhibition when treated with ethylene. Ethylene interacts with GA and auxin pathways to regulate root growth, as well as JA and auxin pathways to regulate root hair formation. Ethylene plays a role in inhibiting stem growth, and can also stimulate stem growth in shaded conditions. | Dugardeyn and Straeten (2008) |
| DWF1, DWF4 | |  |  |  |  |  |
| DWF1, DWF4 | Apple (*Malus domestica*) | *MdDWF1-1*: *MD13G1007700*  *MdDWF1-2*: *MD16G1000400*  *MdDWF4-1*: *MD02G1149000*  *MdDWF4-2*: *MD15G1263900*  *MdDWF4-3*: *MD17G1120200*  (*M. domestica* GDDH13 genome v1.1) | Rooted seedlings:  M.9-T337, Yanfu No. 6 (YF), Nagafu No. 2 (CF), *M. hupehensis*  Rootstock:  M.9, CF  Scion: CF | Phylogeny, synteny analysis, gene expression (qPCR), hormone treatment and quantification | YF trees are spur type and have lower shoot elongation rate, number of internodes, and average internode length relative to CF. *mddwf1-1* and *mddwf1-2* are highly expressed in CF relative to YF. CF/M.9 (dwarf) trees have lower *mddwf4* and *mddwf1* expression relative to CF/CF. | Zheng, L. et al. (2018) |
| DWF4 | Apple (*Malus domestica*) | *MdDWF4*: *MDP0000498540*  (*M. domestica* genome v1.0) | Dwarfing rootstock:  M.9 Pajam 2, M26, GM256, B9 and M.9 T337  Non-dwarfing rootstock:  *M. baccata*, *M. robusta*, *M. sieversii*, *M. prunifolia*, and *M. zumi* | Gene expression (qPCR), *MdWRKY9* overexpression in apple, subcellular localization, transcriptional inhibition assays, hormone quantification, CHIP-qPCR | Overexpression of *MdWRKY9* in apple resulted in significantly lower expression of *mddwf4* and lower brassinosteroid content. *MdWRKY9* binds and inhibits expression of the brassinosteroid biosynthetic gene *MdDWF4* (see above). | Zheng, X. et al. (2018) |
| DWF1, DWF4 | Arabidopsis, Tomato | *AtDWF1*: *At3g19820*  *AtDWF4*: *At3g50660*  (TAIR10) |  | Review of multiple BR biosynthetic genes, pathways, and mutant phenotypes and chemicals that led to these findings | *dwf* mutants exhibit extreme dwarfism, with additional alteration in leaf and inflorescence development. DWARF1 is a biosynthetic enzyme involved in reducing C24 of BR. DWARF4 is a cytochrome P450 enzyme involved in hydroxylating C22 of BR and represents a rate-limiting step in biosynthesis. | Kwon and Choe (2004) |
| IPT3 | |  |  |  |  |  |
| IPT3 | Apple (*Malus domestica*) | *MdPIN1*: Unclear. Degenerate primers designed based on Arabidopsis, lupin, pea, and *Populus* sequences.  *MdIPT3*: Unclear. Degenerate primers designed based on *Malus hupehensis*, Arabidopsis, and cabbage sequences. | Rootstock:  M9 and MM (*M. x micromalus*)  Scion:  Red Fuji  Interstock:  M9 | Gene expression (qPCR), hormone quantification, grafting substitution experiments (rootstock substitutions, rootstock bridging, and bark substitutions) | M9 (common dwarfing rootstock) seedlings showed lower *PIN1* and *IPT3* expression, as well as lower auxin and zeatin across different plant tissues, compared with MM seedlings. Grafting combinations including M9 as rootstock or standard interstock similarly frequently had reduced *PIN1* and *IPT3* expression and reduced hormone levels compared with MM grafting combinations. The reduced levels were largely rescued when MM or Red Fuji were introduced as rootstock substitutions, bridged, or bark substitutions. | Li et al. (2012) |
|  | Arabidopsis | *AtIPT3*: *At3g63110*  *AtIPT5*: *At5g19040*  *AtAHK2*: *At5g35750*  *AtAHK3*: *At1g27320*  *AtAHK4*: *At2g01830*  (TAIR10) | Columbia-0 | Root architecture phenotyping, gene expression (GUS), hormone treatments, overexpression of cytokinin catabolism genes, mutant analysis of biosynthesis genes | Increasing concentrations ot cytokinin led to decreases in lateral root (LR) density. Loss of *IPT3* and other *IPT* (cytokinin biosynthesis) genes resulted in increased LR formation and density. Similarly, loss of *AHK* cytokinin receptor genes led to increased LR densities. | Chang, Ramireddy, and Schmülling, (2013) |
| MAX1, MAX2, MAX3, MAX4 | | |  |  |  |  |
| MAX1, MAX2, MAX3, MAX4 | Apple (*Malus domestica*) | *MdMAX1-1*: *MDP0000130133*  *MdMAX1-2*: *MDP0000677258*  *MdMAX1-3*: *MDP0000909874*  *MdMAX2-1*: *MDP0000466825*  *MdMAX3-1*: *MDP0000197409*  *MdMAX3-2*: *MDP0000139334*  *MdMAX4-1*: *MDP0000227870*  *MdMAX4-4*: Unclear  (Based on primer sequences, *M. domestica* genome v1.0) | Standards:  McIntosh genotypes 21-S, 31-S, and 77-S  Columnar apples: Wijcik genotypes 21-C, 31-C, 77-C | Gene expression (qPCR) comparing standard and columnar phenotypes, strigolactone quantification, overexpression of *MdCo31* in tobacco | *MdMAX* gene expression was frequently higher in columnar apple buds and shoots, as was expression of *MdCo31*. Overexpression of *MdCo31* in tobacco resulted in plants with reduced height, internode lengths, and increase leaf thickness and chlorophyll content. Tobacco *MAX3* was increased in these lines, while tobacco *MAX1* was upregulated only in the line with the shortest stature. | Sun et al. (2020a) |
|  | Peach (*Prunus persica*) | *PpeMAX1*: *Ppa003950m*  *PpeMAX2*: *Ppa002017m*  *PpeMAX3*: *Ppa017865m*  *PpeMAX4*: *Ppa006042m*  (*P. persica* genome v1.0) | Rootstock:  Lovell, Bailey, Tennessee natural  Scion:  Redhaven (standard), Harrow Beauty (standard), Bounty (standard), Crimson Rocket (pillar), Sweet-N-Up (upright) | Gene expression (qPCR), hormone quantification between standard, upright, and pillar growth habits | *PpeMAX3* and *PpeMAX4* expression was higher in in stems following pruning. *PpeMAX3* expression and auxin concentrations were greater in the roots of pillar phenotype. *PpeMAX1-4* expression was intermediate between pillar and standard. Expression of *PpeMAX1*, *PpeMAX2*, and *PpeMAX4* in roots were higher in pillar, but not statistically significant. | Tworkoski, Webb, and Callahan (2015) |
| MAX1, MAX2 | Apple (*Malus spectabilis*, *M. domestica*, and *M. robusta*) | *MsMAX2*: Used primers based on *MdMAX2* (*MD17G1266700*)  *MsMAX1*: Used primers based on *MdMAX1* (*MD15G1057600*)  (*M. domestica* GDDH13 genome v1.1) | Rootstock:  M. robusta  Scion:  More-Branching (*MB*) mutant and wild type (WT) of *M. spectabilis* cultivar Bly114, *M. domestica* cultivars T337, M26, and Fuji Nagafu2 | Morphological and anatomical phenotypes, hormone quantification, gene expression (transcriptome and qPCR) | *MB* mutants had decreased height, increased branch number, and narrower branch angles. *MsMAX1* and *MsMAX2* were upregulated in axillary buds during outgrowth. | Tan et al. (2019) |

- An, H., Luo, F., Wu, T., Wang, Y., Xu, X., Zhang, X., et al. (2017a). Dwarfing Effect of Apple Rootstocks Is Intimately Associated with Low Number of Fine Roots. *Hortscience* 52, 503–512. doi: 10.21273/hortsci11579-16.
- An, J., Liu, X., Li, H., You, C., Shu, J., WANG, X., et al. (2017b). Molecular cloning and functional characterization of *MdPIN1* in apple. *J Integr Agr* 16, 1103–1111. doi: 10.1016/s2095-3119(16)61554-x.
- Bulley, S. M., Wilson, F. M., Hedden, P., Phillips, A. L., Croker, S. J., and James, D. J. (2005). Modification of gibberellin biosynthesis in the grafted apple scion allows control of tree height independent of the rootstock. *Plant Biotechnol J* 3, 215–223. doi: 10.1111/j.1467-7652.2005.00119.x.
- Chang, L., Ramireddy, E., and Schmülling, T. (2013). Lateral root formation and growth of *Arabidopsis* is redundantly regulated by cytokinin metabolism and signalling genes. *J Exp Bot* 64, 5021–5032. doi: 10.1093/jxb/ert291.
- Cheng, J., Ma, J., Zheng, X., Lv, H., Zhang, M., Tan, B., et al. (2021). Functional Analysis of the *Gibberellin 2-*oxidase Gene Family in Peach. *Front Plant Sci* 12, 619158. doi: 10.3389/fpls.2021.619158.
- Cheng, J., Zhang, M., Tan, B., Jiang, Y., Zheng, X., Ye, X., et al. (2019). A single nucleotide mutation in GID1c disrupts its interaction with DELLA1 and causes a GA‐insensitive dwarf phenotype in peach. *Plant Biotechnol J* 17, 1723–1735. doi: 10.1111/pbi.13094.
- Dardick, C., Callahan, A., Horn, R., Ruiz, K. B., Zhebentyayeva, T., Hollender, C., et al. (2013). PpeTAC1 promotes the horizontal growth of branches in peach trees and is a member of a functionally conserved gene family found in diverse plants species. *Plant J* 75, 618–630. doi: 10.1111/tpj.12234.
- Dugardeyn, J., and Straeten, D. V. D. (2008). Ethylene: Fine-tuning plant growth and development by stimulation and inhibition of elongation. *Plant Sci* 175, 59–70. doi: 10.1016/j.plantsci.2008.02.003.
- Foster, T. M., McAtee, P. A., Waite, C. N., Boldingh, H. L., and McGhie, T. K. (2017). Apple dwarfing rootstocks exhibit an imbalance in carbohydrate allocation and reduced cell growth and metabolism. *Hortic Res* 4, 17009. doi: 10.1038/hortres.2017.9.
- Gan, Z., Wang, Y., Wu, T., Xu, X., Zhang, X., and Han, Z. (2018). *MdPIN1b* encodes a putative auxin efflux carrier and has different expression patterns in BC and M9 apple rootstocks. *Plant Mol Biol* 96, 353–365. doi: 10.1007/s11103-018-0700-6.
- Griffiths, J., Murase, K., Rieu, I., Zentella, R., Zhang, Z.-L., Powers, S. J., et al. (2006). Genetic Characterization and Functional Analysis of the GID1 Gibberellin Receptors in Arabidopsis. *Plant Cell Online* 18, 3399–3414. doi: 10.1105/tpc.106.047415.
- Guseman, J. M., Webb, K., Srinivasan, C., and Dardick, C. (2017). DRO1 influences root system architecture in Arabidopsis and *Prunus* species. *Plant J* 89, 1093–1105. doi: 10.1111/tpj.13470.
- Harrison, C. J. (2017). Auxin transport in the evolution of branching forms. *New Phytol* 215, 545–551. doi: 10.1111/nph.14333.
- Hollender, C. A., Hadiarto, T., Srinivasan, C., Scorza, R., and Dardick, C. (2016). A brachytic dwarfism trait (dw) in peach trees is caused by a nonsense mutation within the gibberellic acid receptor PpeGID1c. *New Phytol* 210, 227–239. doi: 10.1111/nph.13772.
- Křeček, P., Skůpa, P., Libus, J., Naramoto, S., Tejos, R., Friml, J., et al. (2009). The PIN-FORMED (PIN) protein family of auxin transporters. *Genome Biol* 10, 249. doi: 10.1186/gb-2009-10-12-249.
- Kwon, M., and Choe, S. (2004). Brassinosteroid biosynthesis and dwarf mutants. *J Plant Biol* 48, 1-15. doi: 10.1007/bf03030559.
- Li, C., Zheng, L., Wang, X., Hu, Z., Zheng, Y., Chen, Q., et al. (2019). Comprehensive expression analysis of *Arabidopsis* GA2-oxidase genes and their functional insights. *Plant Sci* 285, 1-13. doi: 10.1016/j.plantsci.2019.04.023.
- Li, G., Ma, J., Tan, M., Mao, J., An, N., Sha, G., et al. (2016). Transcriptome analysis reveals the effects of sugar metabolism and auxin and cytokinin signaling pathways on root growth and development of grafted apple. *Bmc Genomics* 17, 150. doi: 10.1186/s12864-016-2484-x.
- Li, H. L., Zhang, H., Yu, C., Ma, L., Wang, Y., Zhang, X. Z., et al. (2012). Possible roles of auxin and zeatin for initiating the dwarfing effect of M9 used as apple rootstock or interstock. *Acta Physiol Plant* 34, 235–244. doi: 10.1007/s11738-011-0822-9.
- Ma, Y., Xue, H., Zhang, L., Zhang, F., Ou, C., Wang, F., et al. (2016). Involvement of Auxin and Brassinosteroid in Dwarfism of Autotetraploid Apple (*Malus × domestica*). *Sci Rep-uk* 6, 26719. doi: 10.1038/srep26719.
- Martín‐Rejano, E. M., Camacho‐Cristóbal, J. J., Herrera‐Rodríguez, M. B., Rexach, J., Navarro‐Gochicoa, M. T., and González‐Fontes, A. (2011). Auxin and ethylene are involved in the responses of root system architecture to low boron supply in *Arabidopsis* seedlings. *Physiol Plantarum* 142, 170–178. doi: 10.1111/j.1399-3054.2011.01459.x.
- Pandey, B. K., Huang, G., Bhosale, R., Hartman, S., Sturrock, C. J., Jose, L., et al. (2021). Plant roots sense soil compaction through restricted ethylene diffusion. *Science* 371, 276–280. doi: 10.1126/science.abf3013.
- Qi, L., Chen, L., Wang, C., Zhang, S., Yang, Y., Liu, J., et al. (2020). Characterization of the Auxin Efflux Transporter PIN Proteins in Pear. *Plants* 9, 349. doi: 10.3390/plants9030349.
- Sun, X., Wen, C., Hou, H., Huo, H., Zhu, J., Dai, H., et al. (2020). Genes involved in strigolactone biosyntheses and their expression analyses in columnar apple and standard apple. *Biol Plantarum* 64, 68–76. doi: 10.32615/bp.2019.047.
- Swarup, R., and Bhosale, R. (2019). Developmental Roles of AUX1/LAX Auxin Influx Carriers in Plants. *Front Plant Sci* 10, 1306. doi: 10.3389/fpls.2019.01306.
- Tan, M., Li, G., Chen, X., Xing, L., Ma, J., Zhang, D., et al. (2019). Role of Cytokinin, Strigolactone, and Auxin Export on Outgrowth of Axillary Buds in Apple*. Front Plant Sci* 10, 616. doi: 10.3389/fpls.2019.00616.
- Tworkoski, T., Webb, K., and Callahan, A. (2015). Auxin levels and MAX1–4 and TAC1 gene expression in different growth habits of peach. *Plant Growth Regul* 77, 279–288. doi: 10.1007/s10725-015-0062-x.
- Waite, J. M., and Dardick, C. (2021). The roles of the IGT gene family in plant architecture: past, present, and future. *Curr Opin Plant Biol* 59, 101983. doi: 10.1016/j.pbi.2020.101983.
- Wang, L., Cai, W., Du, C., Fu, Y., Xie, X., and Zhu, Y. (2018). The isolation of the IGT family genes in *Malus × domestica* and their expressions in four idiotype apple cultivars. *Tree Genet Genomes* 14, 46. doi: 10.1007/s11295-018-1258-9.
- Zheng, L., Zhao, C., Mao, J., Song, C., Ma, J., Zhang, D., et al. (2018a). Genome-wide identification and expression analysis of brassinosteroid biosynthesis and metabolism genes regulating apple tree shoot and lateral root growth. *J Plant Physiol* 231, 68–85. doi: 10.1016/j.jplph.2018.09.002.
- Zheng, X., Zhang, H., Xiao, Y., Wang, C., and Tian, Y. (2019). Deletion in the Promoter of PcPIN-L Affects the Polar Auxin Transport in Dwarf Pear (*Pyrus communis* L.). Sci Rep-uk 9, 18645. doi: 10.1038/s41598-019-55195-7.
- Zheng, X., Zhao, Y., Shan, D., Shi, K., Wang, L., Li, Q., et al. (2018b). *MdWRKY9* overexpression confers intensive dwarfing in the M26 rootstock of apple by directly inhibiting brassinosteroid synthetase MdDWF4 expression*. New Phytol* 217, 1086–1098. doi: 10.1111/nph.14891.
